# Integrated analysis of competing endogenous RNA network revealing lncRNAs as potential prognostic biomarkers in human lung squamous cell carcinoma

**DOI:** 10.18632/oncotarget.19627

**Published:** 2017-07-27

**Authors:** Jing Sui, Si-Yi Xu, Jiali Han, Song-Ru Yang, Cheng-Yun Li, Li-Hong Yin, Yue-Pu Pu, Ge-Yu Liang

**Affiliations:** ^1^ Key Laboratory of Environmental Medicine Engineering, Ministry of Education, School of Public Health, Southeast University, Nanjing, P.R. China; ^2^ Department of Epidemiology, Richard M. Fairbanks School of Public Health, Melvin and Bren Simon Cancer Center, Indiana University, Indianapolis, IN, USA; ^3^ Department of Thoracic Surgery, Nanjing Chest Hospital, Nanjing, P.R. China

**Keywords:** lncRNA, ceRNA network, LUSC, prognostic biomarker

## Abstract

Accumulating evidence shows the important role of long non-coding RNAs (lncRNAs) in competing endogenous RNA (ceRNA) networks for predicting survival in tumor patients. However, prognostic biomarkers for lung squamous cell carcinoma (LUSC) are still lacking. The objective of this study is to identify a lncRNA signature for evaluation of overall survival (OS) in 474 LUSC patients from The Cancer Genome Atlas (TCGA) database. A total of 474 RNA sequencing profiles in LUSC patients with clinical data were obtained, providing a large sample of RNA sequencing data, and 83 LUSC-specific lncRNAs, 26 miRNAs, and 85 mRNAs were identified to construct the ceRNA network (fold change>2, P<0.05). Among these above 83 LUSC-specific lncRNAs, 22 were assessed as closely related to OS in LUSC patients using a univariate Cox proportional regression model. Meanwhile, two (FMO6P and PRR26) of the above 22 OS-related lncRNAs were identified using a multivariate Cox regression model to construct a risk score as an independent indicator of the prognostic value of the lncRNA signature in LUSC patients. LUSC patients with low-risk scores were more positively correlated with OS (P<0.001). The present study provides a deeper understanding of the lncRNA-related ceRNA network in LUSC and suggests that the two-lncRNA signature could serve as an independent biomarker for prognosis of LUSC.

## INTRODUCTION

Lung cancer remains one of the most frequently diagnosed and fatal cancers globally. In 2012 nearly 1.8 million new cases were diagnosed, causing 1.6 million deaths worldwide, with a sharp rise from 2008 [1]. Non-small cell lung cancer, including lung squamous cell carcinoma (LUSC) and lung adenocarcinoma (LUAD), is the most pathological type (approximate 80%) in lung cancer. Nearly 30% of NSCLC is LUSC, which causes approximately 400,000 deaths annually worldwide, with both high incidence and poor prognosis (5-year survival rate<15%) [2]. Based on tumor node metastasis (TNM) taxonomy, LUSC can be classified into stages I, II, III, and IV [3]. Recent studies show that LUSC is closely associated with smoking and is more common in men than in women [4]. It is important to distinguish between LUSC and LUAD in the management of NSCLC since their therapeutic regimens and targeted agents differ [5]. Thus, identify effective potential molecular biomarkers for distinguishing between LUSC and LUAD is urgent. In the present study, we aim to find effective potential molecular biomarkers for predicting survival in LUSC.

Long non-coding RNAs (lncRNAs), ranging from 200 nucleotides to 100 kb in length, can modulate gene expression at the transcriptional, post-transcriptional, and epigenetic levels and are broadly distributed in the genome [6–9]. A growing body of evidence demonstrates that lncRNA expression profiles are different in tumors tissues compared to the adjacent non-tumor tissues in various cancers [10–12], including LUSC [13, 14]. It has been proposed that the differentially expressed lncRNAs may correlate with progression and survival in various cancers, which have also been detected in LUSC [15–19].

In 2011, the ceRNA (competing endogenous RNA) hypothesis was presented as a novel regulatory mechanism between non-coding RNA and coding RNA [20]. The central concept is that RNA interacts with miRNA response elements (MREs); this kind of RNA competition crosstalk also exists between lncRNAs and mRNAs [21].

Although numerous lncRNAs have been determined to predict outcomes for lung cancer, the conclusions of previous studies are inconsistent, possibly due to small sample sizes. Recently, lncRNA expression profiles were obtained from The Cancer Genome Atlas (TCGA) database, an open-access and publicly available large-scale database. In the present study, the TCGA database was first used to obtain lncRNA expression profiles and combined with clinical features to construct a lncRNA-miRNA-mRNA ceRNA network in LUSC. Through an integrated analysis of lncRNA expression patterns in the ceRNA network, we identified a lncRNA signature in LUSC with two lncRNAs (FMO6P and PRR26) as a new candidate indicator with the potential to predict overall survival (OS) in LUSC patients.

## RESULTS

### Identification of significantly differentially expressed lncRNAs

**Figure 1:**
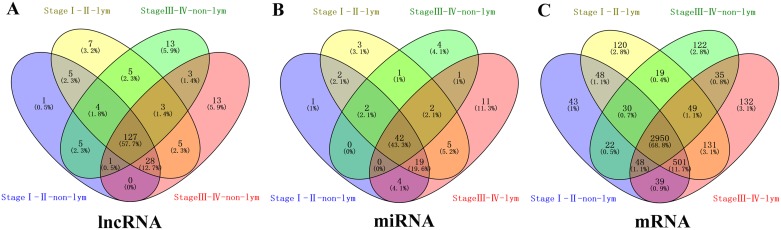
Venn diagram analysis of differentially expressed RNA in LUSC **(A)** lncRNAs; **(B)** miRNAs; **(C)** mRNAs. Lym, lymphatic metastasis; nLym, non- lymphatic metastasis. Each oval represents a group. The brown intersection in the middle represents RNAs, which are consistently and significantly differentially expressed in four groups.

**Figure 2:**
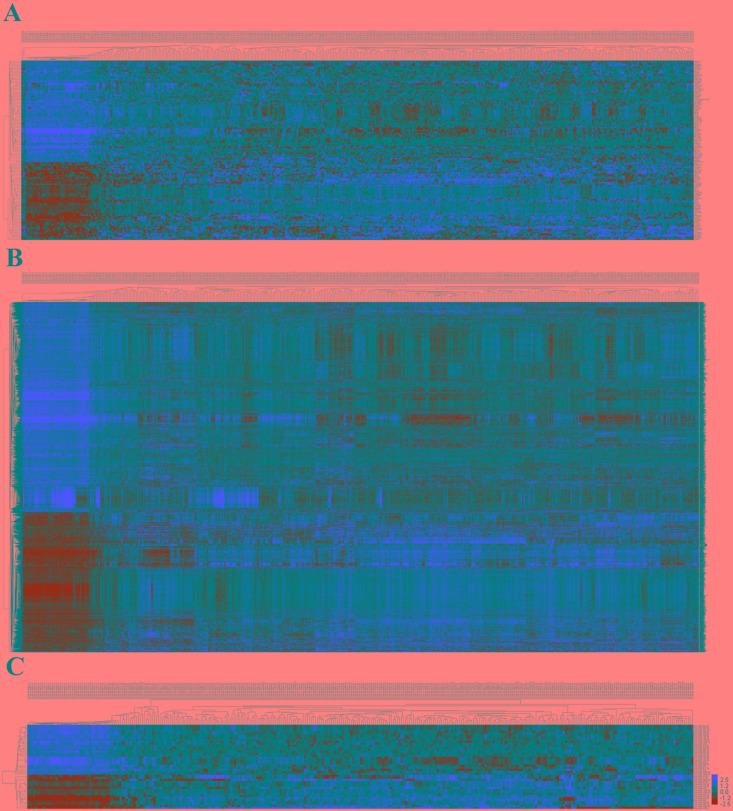
The differential expression of intersected RNAs in LUSC **(A)** lncRNAs; **(B)** miRNAs; **(C)** miRNAs. A heatmap showing the differentially expressed RNAs.

In 474 LUSC patients from TCGA database, we initially performed differential expression analysis by comparing the expression of 1801 lncRNAs in LUSC and adjacent normal lung tissue in the TCGA database. We set fold change>2 and P value>0.05 as cutoffs to identify significantly differentially expressed lncRNAs. Then we obtained 171 differentially expressed lncRNAs between stages I-II (non-lymphatic metastasis) LUSC and adjacent-normal lung tissue, 161 differentially expressed lncRNAs between stages III-IV (non-lymphatic metastasis) LUSC and adjacent-normal lung tissue, 184 differentially expressed lncRNAs between stages I-II (lymphatic metastasis) LUSC and adjacent-normal lung tissue, and 180 differentially expressed lncRNAs between stages III-IV (lymphatic metastasis) LUSC and adjacent-normal lung tissue (fold change>2, P value<0.05). When we combined these four groups of differentially expressed lncRNAs, 127 differentially expressed lncRNAs (55 up-regulated and 72 down-regulated) showed consistently differential expression (Figures 1A and 2A, Supplementary Table 1) and were thus selected to construct the ceRNA network.

### miRNA predicted targets analysis and ceRNA network construction

A total of 1,030 miRNAs and 18,633 mRNAs were identified from the TCGA database, 101 miRNAs and 4,289 mRNAs of which were found to be differentially expressed between LUSC and adjacent normal lung tissue (fold change>2, P<0.05). In total, 42 differentially expressed miRNAs (17 up-regulated and 25 down-regulated) and 2950 differentially expressed mRNAs (1180 up-regulated and 1770 down-regulated) (shown in Figures 1B, 1C and 2B, 2C; Supplementary Tables 2, 3) were selected to construct the ceRNA network.

The next step was to predict the mRNA and lncRNAs targeted by miRNA. We then focused on the targeting relationship between the 42 miRNAs and 2,950 mRNAs as mentioned above. miRTarBase and Targetscan were used to predict miRNA-targeted mRNAs. The result showed that 26 of 42-LUSC specific miRNAs might target to the 85 of 2,950 LUSC-specific mRNAs (Table 1). A few targets are tumor-associated genes such as CDC25A, COL11A1, FZD10, INHBE, ISL1, NAT8L, PAX7, PLAU, SIX4, and TFAP4.

**Table 1:** miRNAs targeting LUSC specific mRNAs

miRNA	mRNA
hsa-miR-130b-3p	ATP1A2, CHRM2, CSF1, IL6ST, LDLR, LRP2, MAGI1, PPARG, ROBO2, S1PR1, TGFBR2
hsa-miR-130b-5p	NRG3, PLCL1
hsa-miR-144-3p	TNFSF11, UCK2
hsa-miR-182-5p	CACNB4, COL4A4, DKK2, EPAS1, MITF, SGMS2, SORT1, ST6GALNAC3, ZFP36
hsa-miR-183-5p	C8B, NTN4, PRKCB, ZEB1
hsa-miR-196a-5p	AQP4, COL14A1
hsa-miR-196b-5p	COL14A1
hsa-miR-205-5p	ACACB, ERBB4, MAGI1, MAGI3, RAB11FIP1, SHROOM3
hsa-miR-210-3p	GPD1L
hsa-miR-218-5p	BRCA1, GNG4, HOXA10, UGT8
hsa-miR-3065-3p	COL11A1, COL7A1, GRM4, ISL1, PLAU
hsa-miR-30a-3p	FANCI, SIX4
hsa-miR-30a-5p	GCLC
hsa-miR-30c-2-3p	INHBE, NAT8L, PAX7
hsa-miR-30d-3p	SIX4
hsa-miR-30d-5p	CCNE2
hsa-miR-31-5p	FZD4, LATS2, PRKCE, RORC
hsa-miR-326	FANCE
hsa-miR-490-3p	ADCY10
hsa-miR-497-5p	CDC25A, FZD10, PAX7, TFAP4
hsa-miR-511-5p	CACNA1B, FZD10
hsa-miR-629-3p	COL4A4, DHH, IL6R, KCNJ5, NEGR1, SLC34A2, ZEB1
hsa-miR-708-3p	FGR, SHROOM4
hsa-miR-708-5p	CNTFR, MASP1
hsa-miR-9-5p	ANK2, CNTFR, GABRB2, ID4, IL6R, LIFR, MEF2C, SGMS2, SHC3, SHROOM4
hsa-miR-96-5p	CACNA1C, CACNA2D2, CACNB4, COL13A1, HBEGF, MAGI1, MAGI3, NR4A3, NTN4, SCNN1G, SLC1A1, SLC26A9, SORT1

Subsequently, the targeting relationships among 127 differentially expressed lncRNAs (Supplementary Table 1) and 26 differentially expressed miRNAs (Table 1) were assessed. In the ceRNA network, lncRNA might interact with the miRNA through MREs. Using miRanda to identify the potential MREs, the result showed these 26 LUSC-specific miRNAs might target to 83 of the 127 LUSC-specific lncRNAs (Table 2).

**Table 2:** miRNAs that may target LUSC specific lncRNAs

lncRNA	miRNA
ABCC13	hsa-miR-130b-3p
ABCC6P1	hsa-miR-196b-5p, hsa-miR-31-5p, hsa-miR-96-5p
ABHD11-AS1	hsa-miR-182-5p
AFAP1-AS1	hsa-miR-511-5p
ALOX12P2	hsa-miR-3065-3p, hsa-miR-30a-3p, hsa-miR-30d-3p, hsa-miR-326, hsa-miR-497-5p
C1orf140	hsa-miR-182-5p, hsa-miR-196a-5p, hsa-miR-9-5p, hsa-miR-96-5p
C1orf220	hsa-miR-3065-3p, hsa-miR-30d-3p, hsa-miR-511-5p
CCL15-CCL14	hsa-miR-205-5p
CHIAP2	hsa-miR-182-5p, hsa-miR-708-3p
CMAHP	hsa-miR-205-5p, hsa-miR-629-3p, hsa-miR-708-3p, hsa-miR-708-5p
CYP1B1-AS1	hsa-miR-130b-3p, hsa-miR-708-3p
CYP2B7P	hsa-miR-130b-3p, hsa-miR-708-5p
CYP2D7	hsa-miR-3065-3p
CYP4Z2P	hsa-miR-96-5p
DDX12P	hsa-miR-497-5p
DGCR5	hsa-miR-326, hsa-miR-490-3p
DIRC3	hsa-miR-3065-3p, hsa-miR-30a-3p
DLX6-AS1	hsa-miR-30c-2-3p
DNM1P46	hsa-miR-205-5p
FAM86JP	hsa-miR-218-5p
FAR2P1	hsa-miR-3065-3p, hsa-miR-30c-2-3p, hsa-miR-497-5p
FER1L4	hsa-miR-3065-3p, hsa-miR-30c-2-3p, hsa-miR-490-3p
FIRRE	hsa-miR-30a-3p, hsa-miR-30d-3p
FLJ34503	hsa-miR-130b-5p, hsa-miR-9-5p
FMO6P	hsa-miR-30a-3p
GGTA1P	hsa-miR-9-5p
GVINP1	hsa-miR-196a-5p, hsa-miR-205-5p, hsa-miR-9-5p
HOTAIR	hsa-miR-30a-5p, hsa-miR-326
KC6	hsa-miR-326
KIAA0087	hsa-miR-708-5p, hsa-miR-9-5p
KRTAP5-AS1	hsa-miR-130b-5p
KTN1-AS1	hsa-miR-30c-2-3p, hsa-miR-511-5p
LHFPL3-AS2	hsa-miR-205-5p
LINC00092	hsa-miR-629-3p
LINC00173	hsa-miR-30c-2-3p
LINC00261	hsa-miR-130b-5p, hsa-miR-182-5p, hsa-miR-196a-5p, hsa-miR-629-3p, hsa-miR-708-5p
LINC00312	hsa-miR-708-5p
LINC00319	hsa-miR-326, hsa-miR-497-5p
LINC00341	hsa-miR-205-5p, hsa-miR-708-5p
LINC00472	hsa-miR-205-5p, hsa-miR-96-5p
LINC00482	hsa-miR-196a-5p
LINC00704	hsa-miR-511-5p
LINC00887	hsa-miR-490-3p
LINC00908	hsa-miR-130b-3p, hsa-miR-196b-5p
LINC00924	hsa-miR-196a-5p
LINC00961	hsa-miR-96-5p
LINC00982	hsa-miR-130b-3p
LINC01105	hsa-miR-130b-3p, hsa-miR-182-5p, hsa-miR-183-5p, hsa-miR-629-3p, hsa-miR-708-5p, hsa-miR-96-5p
LOC100499484-C9ORF174	hsa-miR-130b-5p, hsa-miR-708-5p, hsa-miR-96-5p
LOC148709	hsa-miR-3065-3p, hsa-miR-326, hsa-miR-497-5p
LOC285629	hsa-miR-30c-2-3p
LOC399815	hsa-miR-30a-3p, hsa-miR-30d-3p
LOC642846	hsa-miR-3065-3p
LOC90246	hsa-miR-130b-5p
LOC93429	hsa-miR-3065-3p, hsa-miR-30c-2-3p, hsa-miR-326
MEIS3P1	hsa-miR-130b-5p
MGC27382	hsa-miR-130b-5p
MIR31HG	hsa-miR-30c-2-3p, hsa-miR-511-5p
MIR9-3HG	hsa-miR-144-3p, hsa-miR-326, hsa-miR-511-5p
MIR99AHG	hsa-miR-182-5p, hsa-miR-196a-5p, hsa-miR-31-5p
MSL3P1	hsa-miR-30c-2-3p
NAPSB	hsa-miR-205-5p
NCF1B	hsa-miR-182-5p
OR7E91P	hsa-miR-218-5p, hsa-miR-490-3p
PART1	hsa-miR-3065-3p, hsa-miR-30d-3p, hsa-miR-490-3p, hsa-miR-497-5p
PGM5P2	hsa-miR-182-5p, hsa-miR-196a-5p, hsa-miR-9-5p
PRR26	hsa-miR-130b-5p, hsa-miR-96-5p
PSMG3-AS1	hsa-miR-130b-5p, hsa-miR-205-5p, hsa-miR-629-3p, hsa-miR-708-5p, hsa-miR-9-5p
PVT1	hsa-miR-326
PWARSN	hsa-miR-196b-5p, hsa-miR-31-5p
SCARNA12	hsa-miR-3065-3p
SFTA1P	hsa-miR-9-5p
SLC6A10P	hsa-miR-3065-3p, hsa-miR-326, hsa-miR-497-5p
SNHG4	hsa-miR-3065-3p, hsa-miR-497-5p
SOX2-OT	hsa-miR-30a-3p, hsa-miR-30d-3p, hsa-miR-490-3p
TCAM1P	hsa-miR-30c-2-3p, hsa-miR-326, hsa-miR-511-5p
TMPO-AS1	hsa-miR-326
TPRXL	hsa-miR-30c-2-3p
TPTEP1	hsa-miR-708-5p
TRHDE-AS1	hsa-miR-9-5p
UMODL1-AS1	hsa-miR-210-3p
WDFY3-AS2	hsa-miR-31-5p
WWC2-AS2	hsa-miR-130b-5p, hsa-miR-205-5p

**Figure 3:**
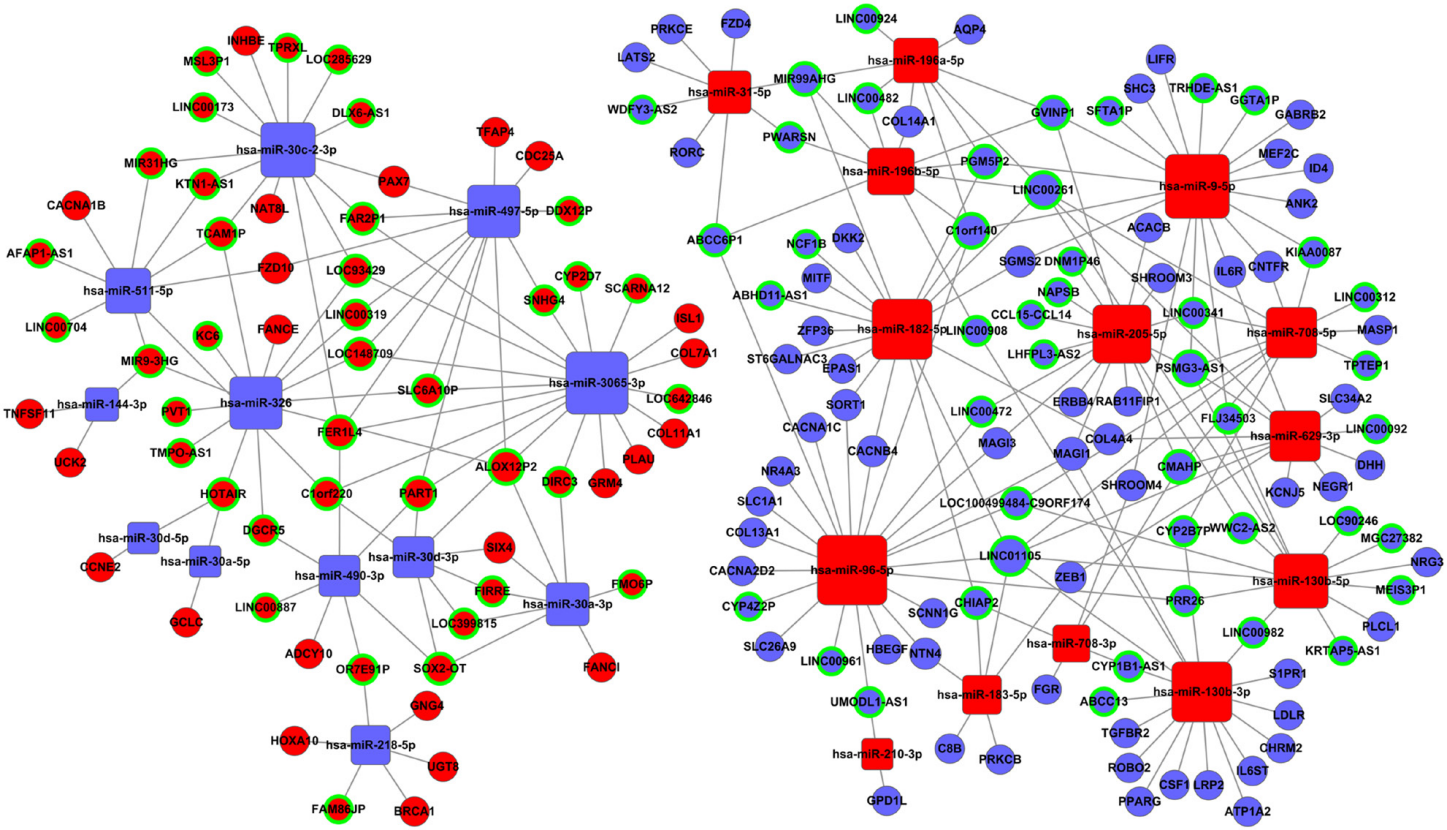
The lncRNA-miRNA-mRNA ceRNA network Blue balls surrounded by green rings = down-regulated lncRNAs; red balls surrounded by green rings = up-regulated lncRNAs; blue diamonds = down-regulated miRNAs; red diamonds = up-regulated miRNAs; blue balls = down-regulated mRNAs; red balls = up-regulated mRNAs.

Based on the above data (Tables 1 and 2), we constructed the lncRNA-miRNA-mRNA ceRNA network with Cytoscape 3.0. To obtain the most reliable results, a maximal information coefficient (MIC) algorithm was employed to screen the pair-wise relationships based on the expression levels of the lncRNA, miRNA, and mRNA in the TCGA database (MIC>0.15, MIC-P^2^>0.15) [22]. In all, 83 lncRNAs, 26 miRNAs, and 85 mRNAs were involved in the ceRNA network (Figure 3).

### Functional assessment

**Figure 4:**
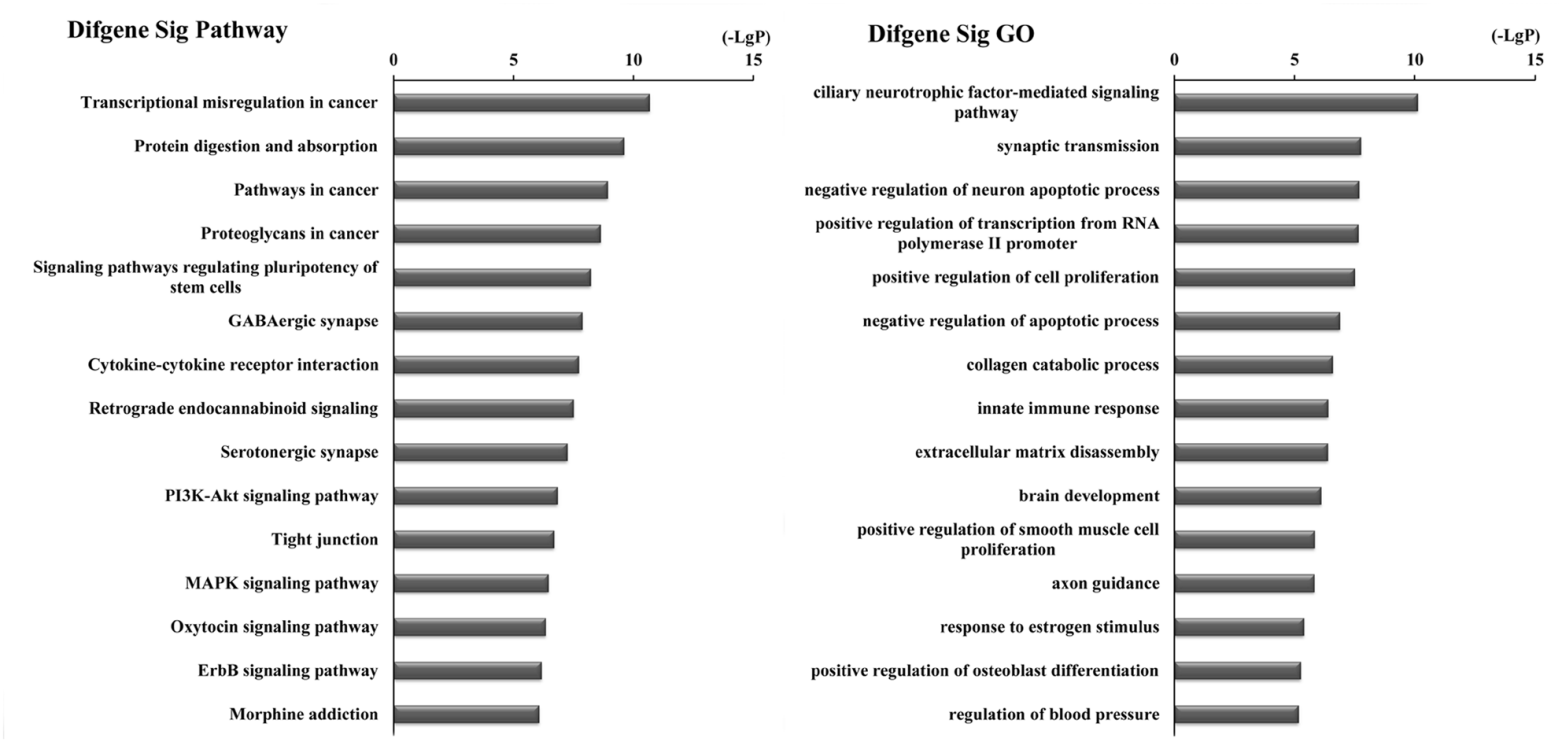
Top 15 enrichment of KEGG pathways and GO terms for differentially expressed mRNAs in ceRNA network

Furthermore, to identify functions associated with the differentially expressed mRNAs in the ceRNA network, we analyzed these mRNAs with DAVID bioinformatics resources. This process revealed enrichment of 81 KEGG pathways and 433 GO terms (P-value<0.05 and enrichment score>2) (Supplementary Table 4). KEGG pathway analyses identified the most significant pathways as Transcriptional dysregulation in cancer (path ID: 05202) and Protein digestion and absorption (path ID: 04974) (Figure 4, Table 3). Five of the top 15 pathways were tumor-related pathways including Transcriptional dysregulation in cancer, Pathways in cancer, Proteoglycans in cancer, PI3K-Akt signaling pathway, and MAPK signaling pathway. There were also tumor-related pathways such as microRNAs in cancer, Wnt signaling pathway, Hippo signaling pathway, NF-kappa B signaling pathway, Ras signaling pathway, Basal cell carcinoma, and Glioma and Small cell lung cancer in the remaining significantly differentially changed pathways. The top GO terms were Ciliary neurotrophic factor-mediated signaling pathway (GO: 0070120) and Synaptic transmission (GO: 0007268) (Figure 4, Table 3).

**Table 3:** Top 15 KEEG pathways and GO terms enriched by the coding genes involved in ceRNA network

Category	Term	No. of genes	-lgP
KEGG pathways	Transcriptional misregulation in cancer	9	10.674
	Protein digestion and absorption	7	9.609
	Pathways in cancer	10	8.926
	Proteoglycans in cancer	8	8.629
	Signaling pathways regulating pluripotency of stem cells	7	8.210
	GABAergic synapse	6	7.868
	Cytokine-cytokine receptor interaction	8	7.720
	Retrograde endocannabinoid signaling	6	7.506
	Serotonergic synapse	6	7.235
	PI3K-Akt signaling pathway	8	6.832
	Tight junction	6	6.693
	MAPK signaling pathway	7	6.457
	Oxytocin signaling pathway	6	6.344
	ErbB signaling pathway	5	6.160
	Morphine addiction	5	6.062
GO	ciliary neurotrophic factor-mediated signaling pathway	4	10.121
	synaptic transmission	9	7.745
	negative regulation of neuron apoptotic process	6	7.667
	positive regulation of transcription from RNA polymerase II promoter	11	7.644
	positive regulation of cell proliferation	9	7.490
	negative regulation of apoptotic process	9	6.882
	collagen catabolic process	5	6.575
	innate immune response	9	6.384
	extracellular matrix disassembly	5	6.371
	brain development	6	6.0965
	positive regulation of smooth muscle cell proliferation	4	5.831
	axon guidance	7	5.819
	response to estrogen stimulus	4	5.376
	positive regulation of osteoblast differentiation	4	5.248
	regulation of blood pressure	4	5.158

### Construction of the LUSC-specific lncRNA-based prognostic signature

The 83 specific lncRNAs in the ceRNA network were further analyzed according to clinical features including gender, tumor stage, TNM staging system, lymph node metastasis, and patient outcome assessment at diagnosis in TCGA database. There were 25 LUSC-specific lncRNAs, the expression levels of which were significantly differentially expressed in clinical feature comparisons (P<0.05; Table 4). Seventeen lncRNAs were differentially expressed in gender, three in tumor stage, three in TNM staging system, six in lymphatic metastasis, and four were differentially expressed in patient outcome assessment.

**Table 4:** The correlations between LUSC specific lncRNAs from ceRNA network and clinical features

Comparisons	Down-regulated	Up-regulated
Gender (Female *vs*. Male)	CMAHP, LINC01105, LINC00261, NAPSB, UMODL1-AS1, KRTAP5-AS1, KRTAP5-AS1, GVINP1, MIR99AHG	LOC399815, KTN1-AS1, TPRXL, KC6, SLC6A10P, LINC00173, DDX12P, FAR2P1
Tumor stage (I-II *vs*. III-IV)		TCAM1P, KTN1-AS1, CYP2D7
TNM staging system (T1 + T2 *vs*. T3 + T4)	CMAHP	KTN1-AS1, TCAM1P
Lymphatic metastasis (No *vs*. Yes)	CYP2B7P, PGM5P2, LOC100499484-C9ORF174	LOC399815, TMPO-AS1, PART1
Patient outcome assessment (Dead *vs*. Alive)	MIR99AHG, PRR26, CCL15-CCL14	CYP2D7

**Figure 5:**
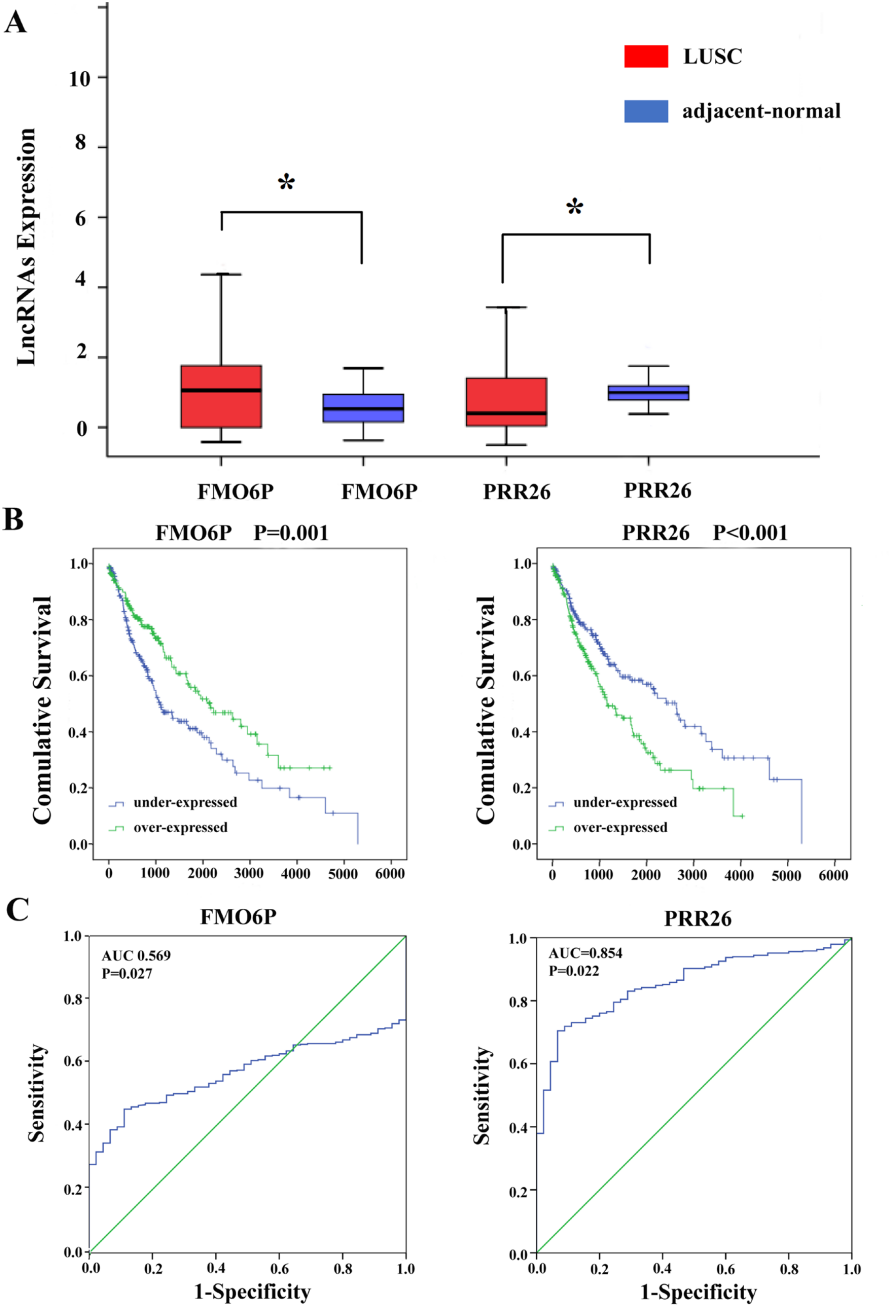
Two differentially expressed lncRNAs (FMO6P and PRR26) **(A)** The expression levels of two lncRNAs in the LUSC tissues compared with adjacent normal tissues. **(B)** Kaplan-Meier curves showing the relationship between the two lncRNAs and OS. The cases were divided into under- and over-expression groups by the mean lncRNAs level. **(C)** ROC curves of the two lncRNAs to distinguish LUSC tissue from adjacent normal tissues.

Univariate Cox proportional hazards regression showed that 22 of the 83 differentially expressed lncRNAs in the ceRNA network were identified to have a significant prognostic value (Table 5). A multivariate Cox proportional hazards regression analysis indicated that only two lncRNAs showed a significant prognostic value for LUSC: KRTAP5-AS1 and SOX2-OT (Figure 5). The risk score for predicting the OS was constructed with the formula: Risk score=exp_FMO6P_ * (-0.401) + exp_PRR26_ * 0.399.

**Table 5:** Prognostic value of the differentially expressed lncRNAs by univariate cox regression analysis

LncRNA	Estimate	StdErr	ChiSq	P	Hazard ratio ( 95%CI)
ABCC13	0.367	0.153	5.738	0.017*	1.444 (1.069-1.950)
ABHD11-AS1	-0.305	0.153	0.3980	0.046*	0.737 (0.546-0.995)
C1orf220	-0.315	0.154	4.208	0.040*	0.729 (0.504-0.986)
CCL15-CCL14	0.363	0.153	5.620	0.018*	1.437 (1.065-1.939)
CHIAP2	0.459	0.154	8.863	0.003*	1.583 (1.170-2.141)
DDX12P	-0.320	0.154	4.349	0.037*	0.726 (0.537-0.981)
DIRC3	-0.437	0.153	8.137	0.004*	0.646 (0.478-0.872)
FMO6P	-0.486	0.154	10.041	0.002*	0.615 (0.455-0.831)
KC6	-0.306	0.153	4.016	0.045*	0.736 (0.546-0.993)
KIAA0087	0.494	0.154	10.348	0.001*	1.639 (1.213-2.214)
KRTAP5-AS1	0.409	0.154	7.089	0.008*	1.505 (1.114-2.035)
KTN1-AS1	-0.485	0.154	9.923	0.002*	0.616 (0.455-0.833)
LINC00261	0.390	0.154	6.397	0.011*	1.478 (1.092-2.000)
LINC00472	-0.299	0.152	3.859	0.049*	0.742 (0.550-0.999)
LINC00961	0.435	0.154	8.002	0.005*	1.545 (1.143-2.088)
PART1	-0.466	0.153	9.269	0.002*	0.627 (0.465-0.847)
PRR26	0.505	0.155	10.657	0.001*	1.657 (1.224-2.244)
PVT1	-0.334	0.152	4.803	0.028*	0.716 (0.531-0.965)
SOX2-OT	-0.473	0.154	9.487	0.002*	0.623 (0.461-0.842)
TCAM1P	0.366	0.153	5.689	0.017*	1.442 (1.067-1.948)
TMPO-AS1	-0.370	0.153	5.828	0.016*	0.691 (0.512-0.933)
UMODL1-AS1	0.302	0.153	3.905	0.048*	1.352 (1.002-1.824)

*: P<0.05.

**Figure 6:**
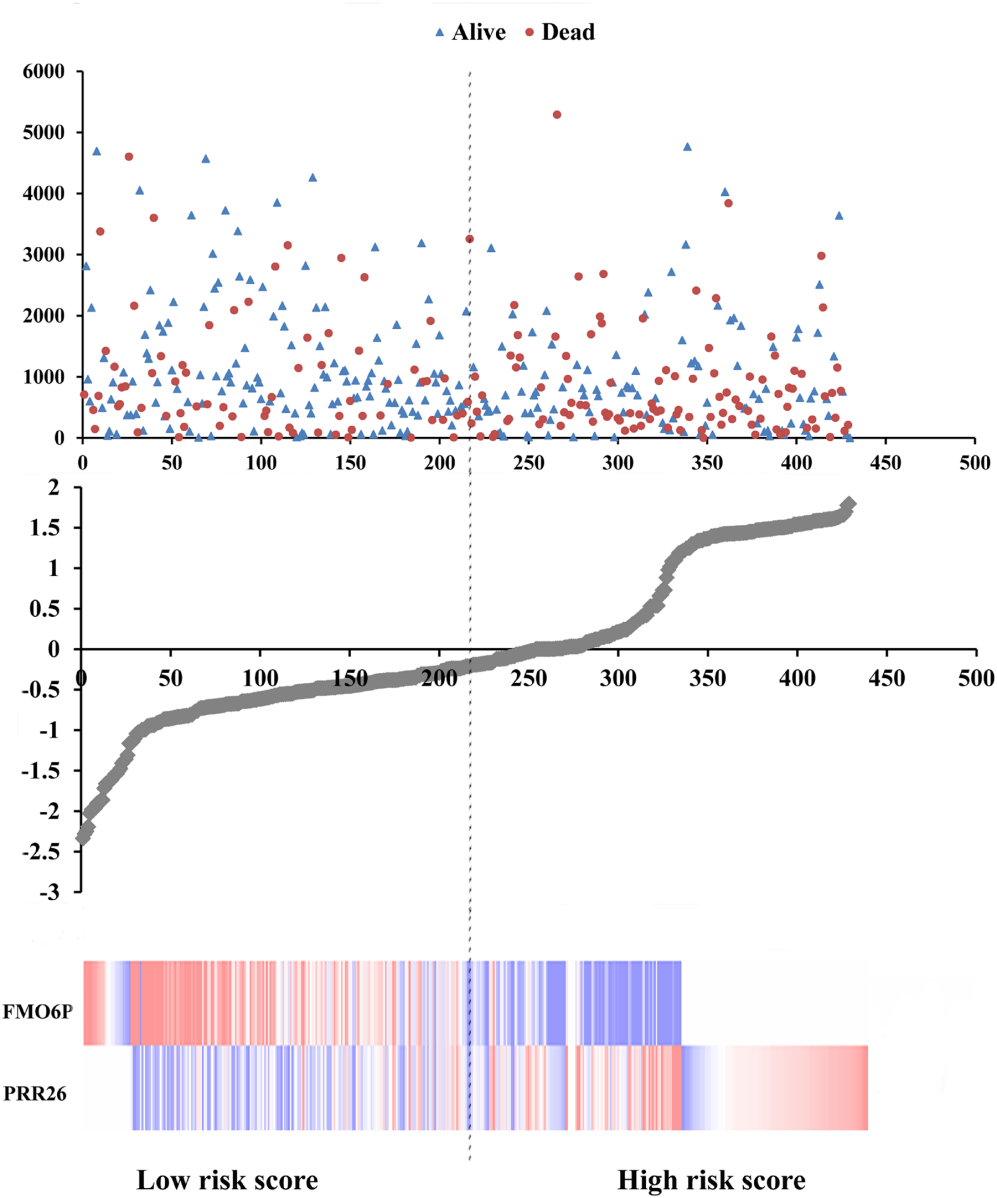
Risk score analysis of the differentially expressed lncRNA signature of LUSC Survival status and duration of cases (Top); risk score of lncRNA signature (Middle); low and high score groups for the 2 lncRNAs (Bottom).

**Figure 7:**
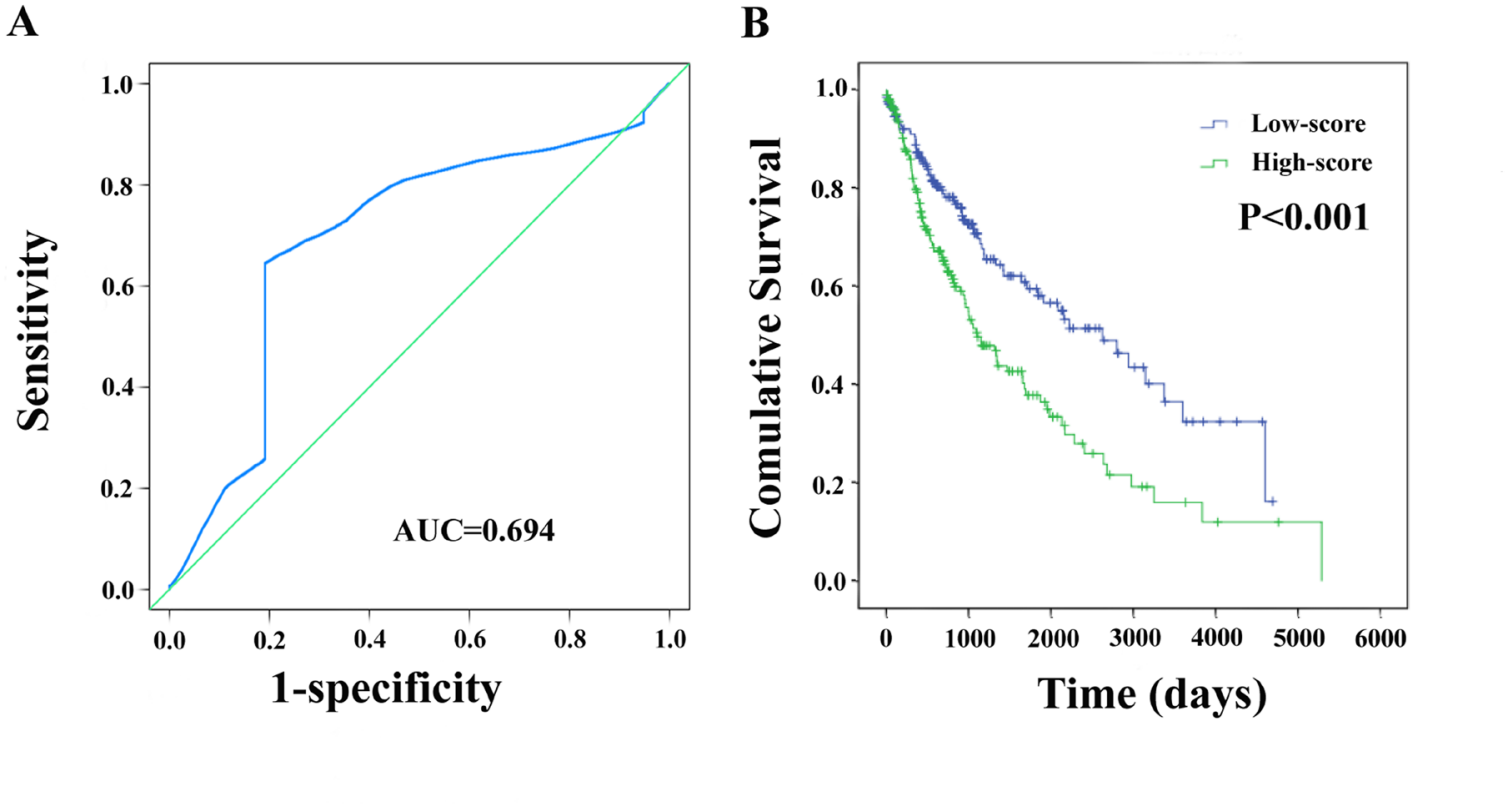
The two-differentially expressed lncRNA signature of LUSC for outcome **(A)** The risk score shown by the time-dependent ROC curve for predicting 5-year survival. **(B)** The Kaplan-Meier test of the risk score for the OS.

Meanwhile, based on the individual inflection point of the prognostic risk score, LUSC patients were divided into low-risk and high-risk groups (Figure 6). The risk score widely predicted 5-year survival of LUSC patients, as the area under ROC curve (AUC) was 0.694 (Figure 7A). Furthermore, K-M curves confirmed that low-risk was more positively correlated with OS (P<0.001, Figure 7B).

**Figure 8:**
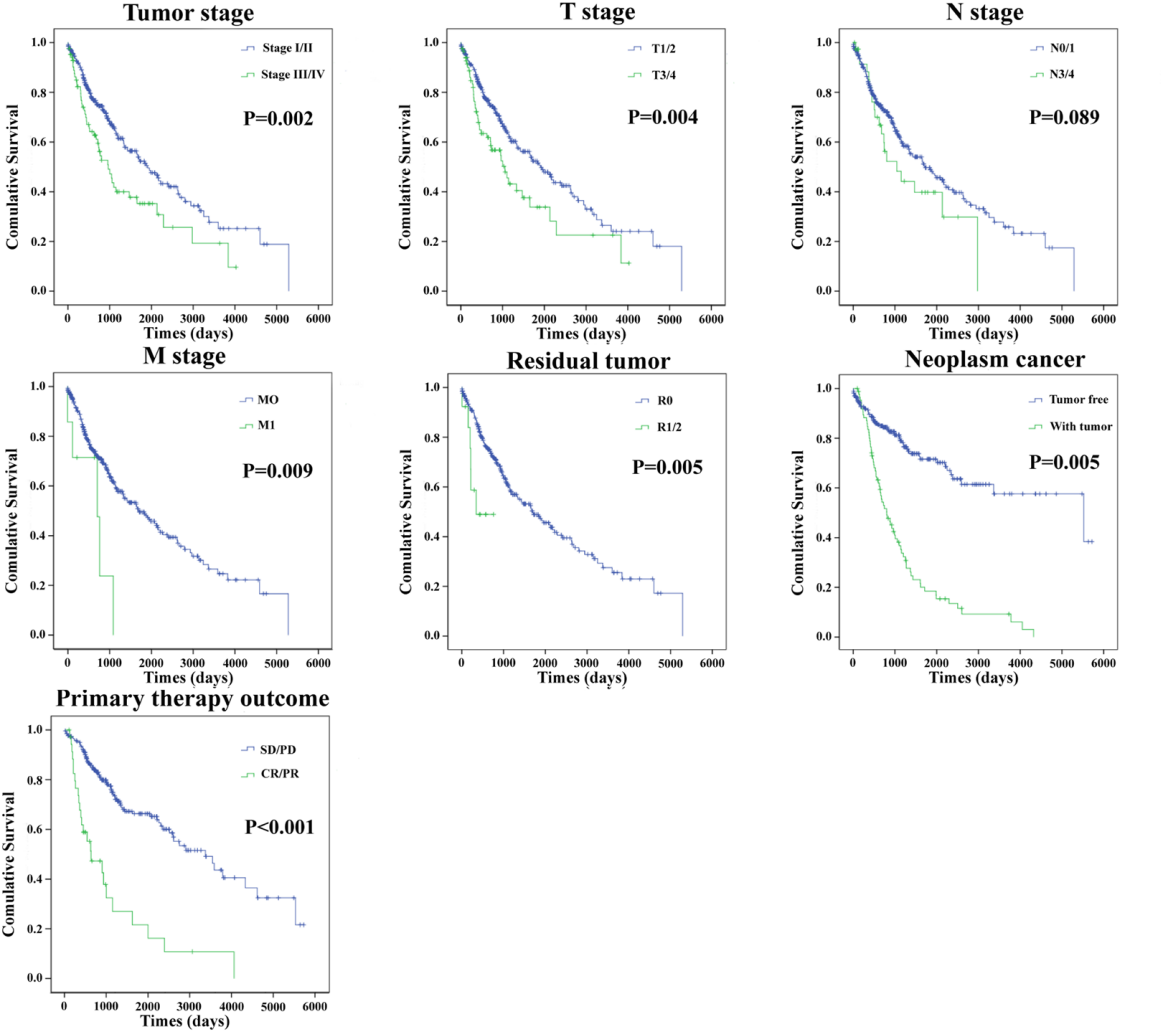
The prognostic value of different clinical features for OS of LUSC patients Kaplan-Meier curves of seven independent prognostic indictors. SD, stable disease; PD, progressive disease; CR, complete remission; PR, partial response.

Moreover, the prognostic value of different clinical features was also evaluated. The univariate Cox proportional hazards regression showed that some clinical features predicted poorer survival of patients with LUSC (Table 6).However, when analyzed by a multivariate Cox regression model, gender, cancer neoplasms, and risk score were the independent prognostic indictors of LUSC (Table 6). The Kaplan-Meier curves of the above clinical features are shown in Figure 8. The results revealed that Tumor stage, T stage, M stage, residual tumor, cancer neoplasm, and primary therapy outcome were associated with OS (P=0.002, P=0.004, P=0.009, P=0.005, P=0.005, and P<0.001, respectively).

**Table 6:** The predictive values of related clinical features and risk score

Variables		Patient N=429	Univariate analysis		Multivariate analysis	
			HR(95% CI)	P	HR(95% CI)	P
Race	White	292	1(reference)		1(reference)	
	Asian	29	1.414(0.859-2.327)	0.173	1.061(0.589-1.912)	0.844
Gender	Female	111	1(reference)		1(reference)	
	Male	311	1.235(0.863-1.766)	0.249	2.663(1.296-5.471)	0.008*
Age	<=65	248	1(reference)		1(reference)	
	>65	169	1.344(0.983-1.838)	0.086	0.996(0.588-1.689)	0.989
Tumor stage	I	202	1(reference)		1(reference)	
	II	135	1.015(0.710-1.452)	0.933	1.225(0.591-2.541)	0.585
	III	78	1.610(1.106-2.343)	0.013*	0.474(0.181-1.242)	0.129
	IV	7	3.464(1.393-8.613)	0.007*	0.966(0.213-4.381)	0.964
T stage	T1	95	1(reference)		1(reference)	
	T2	243	1.255(0.849-1.855)	0.255	0.859(0.473-1.558)	0.617
	T3	63	1.771(1.081-2.899)	0.023*	1.830(0.832-4.028)	0.133
	T4	21	2.470(1.302-4.687)	0.006*	1.865(0.580-6.001)	0.296
N stage	N0	266	1(reference)		1(reference)	
	N1	116	1.080(0.770-1.514)	0.656	1.186(0.454-3.097)	0.728
	N2	34	1.378(0.834-2.277)	0.211	0.707(0.098-5.086)	0.731
	N3	5	2.713(0.665-11.067)	0.164		
M stage	M0	415	1(reference)			
	M1	7	3.119(1.274-7.633)	0.013*		
Atomic neoplasm subdivision	Left lung	179	1(reference)			
	Right lung	220	1.050(0.773-1.426)	0.754	0.868(0.499-1.509)	0.617
	Bronchia	9	0.395(0.097-1.609)	0.195	1.760(0.167-18.498)	0.638
Primary therapy outcome	Complete remission	259	1(reference)		1(reference)	
	Stable disease	14	3.166(1.523-6.579)	0.002*	2.881(0.908-9.143)	0.073
	Progressive disease	22	4.901(2.876-8.351)	<0.001*	1.778(0.829-3.814)	0.139
	Partial remission	6	3.579(1.304-9.823)	0.013*	0.747(0.167-3.336)	0.702
Radiotherapy	NO	318	1(reference)		1(reference)	
	YES	45	1.427(0.915-2.225)	0.117	0.818(0.321-2.084)	0.674
Neoplasm cancer	Tumor free	276	1(reference)		1(reference)	
	With tumor	87	4.229(2.994-5.976)	<0.001*	3.116(1.884-5.154)	<0.001*
Residual tumor	R0	345	1(reference)		1(reference)	
	R1+R2	13	3.135(1.361-7.224)	0.007*	1.561(0.198-12.338)	0.673
Risk score	Low	212	1(reference)		1(reference)	
	High	210	1.815(1.341-2.456)	<0.001*	3.116(1.884-5.154)	0.001*

HR: hazard ratio; CI: confidence interval; *: P<0.05.

**Figure 9:**
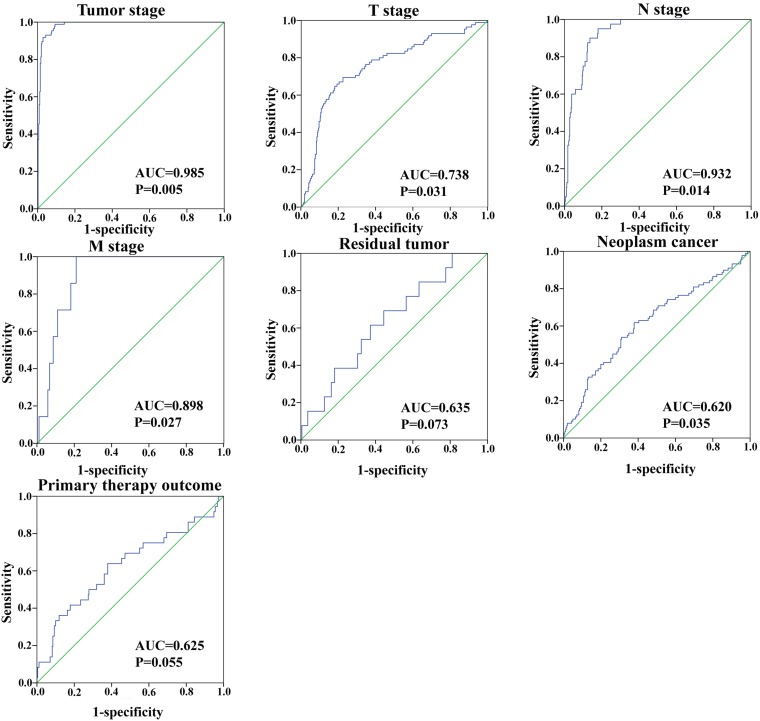
The predictive value of the risk score for clinical features ROC curve predicting different clinical features.

**Figure 10:**
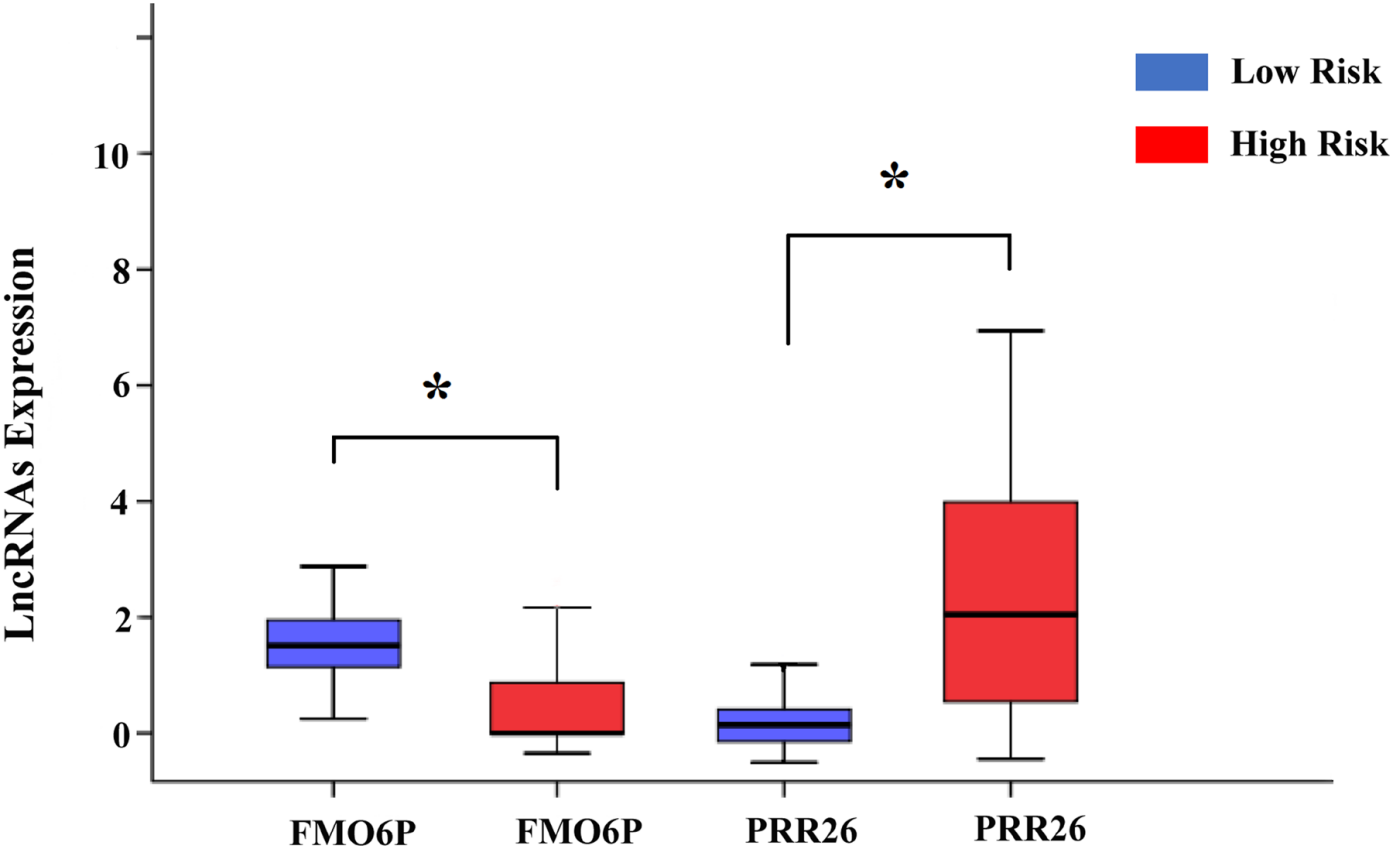
The expression level of the two lncRNAs in the low- and high-risk groups The difference in the expression level of FMO6P and PRR26 between the low-risk and high-risk groups. *P<0.05.

In addition, we assessed the relationship between the risk score based on the differentially expressed lncRNAs signature and various clinical features, and the risk score showed prognostic value for predicting the status of tumor stage, T stage, metastasis, and neoplasm (Figure 9). The expression patterns of these two differentially expressed lncRNAs in the low-score and high-score groups were given in Figure 10.

## DISCUSSION

To define lncRNAs significantly related to OS, we constructed a lncRNA-miRNA-mRNA ceRNA network according to the specific criteria in a large sample of LUSC patients, based on information from the TCGA database. A univariate Cox proportional hazards regression with the significance level set at 0.05 was first performed on 83 differentially expressed lncRNAs from the ceRNA network. A total of 22 lncRNAs were identified as associated with OS. A multivariate Cox proportional hazards regression analysis indicated that FMO6P and PRR26 showed a significant prognostic value for LUSC patients’’ survival. We then developed a risk score by combining the two lncRNAs and found that this two-lncRNA signature independently predicted OS in LUSC patients, which was further validated in LUSC patients. To our knowledge, this is the first study combining a ceRNA network with TCGA data to assess the survival of LUSC patients by constructing a lncRNA-related risk score.

Lung cancer produces the most lethal solid malignancies, and the role of lncRNAs in the genesis and development— and thus diagnosis and prognosis —of lung cancer has been widely studied [23–26]. In addition to the metastatic behavior of lung cancer, lack of precise and accurate biomarkers for diagnosis and prognosis may also contribute strongly to the low survival rate (< 15%). Therefore, there is a pressing need for potential and reliable prognostic biomarkers that predict lung cancer outcomes. In previous studies, expression of miRNA and mRNA have been examined to reveal characteristics associated with lung cancer outcome [27–30]. Differential expression of lncRNAs has rapidly emerged in various studies of tumorigenesis and cancer progression, and the dysregulated lncRNAs play key roles in cancer prognosis.

There is accumulating evidence that lncRNAs might be a pivotal component in the ceRNA network by modulating other RNA transcripts [31–33]. CHIAP2 was detected in the lung cancer-related ceRNA network [34]. Snhg1 promoted cell proliferation by acting as a sponge for the tumor suppressor miR-338 in esophageal cancer cells [35]. Wang et al. [36] affirmed that TUG1 might affect ROCK1 expression, which could mediate migration/invasion to influence prognosis, by working as a ceRNA manner by targeting miR-335-5p. The up-regulation of AFAP1-AS1 was associated with poor prognosis in NSCLC patients [37]. Therefore, potential connections among lncRNA, miRNA, and mRNA might exist in the genesis and development of LUSC. In the present study, we first used the TCGA database to construct a ceRNA network to reveal a novel regulatory network in LUSC. Some cancer-specific lncRNAs, such as ABCC13, HOTAIR, LINC00472, and FER1L4 have also been reported to act as potential diagnostic and prognostic biomarkers in cancers [38–41]. In the present study, we sought to determine whether the LUSC-specific lncRNAs in the ceRNA network were indirectly related to mRNA signal pathways. The results of this pathway analysis showed that there were a few pathways related to cancer. Hence, our results suggest that specific lncRNAs may play crucial roles in the genesis and development of LUSC.

Based on public data from large-scale samples, the prognostic value of lncRNAs has been evaluated in various cancers, including lung adenocarcinoma [42], gastric cancer [43], urothelial carcinoma [44], colorectal cancer [45], intrahepatic cholangiocarcinoma [46], and breast cancer [47]. One recent study evaluated the prognostic value of miRNAs and mRNAs in LUSC. Using the TCGA database, Gao et al. [48] found that 12 of the 133 most significantly altered miRNAs were associated with OS of LUSC After a comprehensive analysis, a seven-miRNA signature for prediction of OS in LUSC patients was established. Similarly, multivariable Cox regression analysis indicated that up-regulated expression of ASCL2 was an independent prognostic factor for OS (HR=2.764, P<0.05) in LUSC patients [49]. However, the relationship between differentially expressed lncRNAs and survival in LUSC patients has not been studied in large-scale samples using comprehensive analysis. Unlike previous studies, we combined the ceRNA network with TCGA data to analyze the lncRNAs in LUSC. Since then, we determined that significantly differential expression of two novel lncRNAs (FMO6P and PRR26) could be a novel independent risk factor for LUSC. Moreover, the risk score based on these two lncRNAs could be a new indicator for the prognosis of LUSC patients.

However, there is no report on the association between these two lncRNAs and disease. Moreover, no study has investigated the function of the above two lncRNAs. Here, we constructed a lncRNA-miRNA-mRNA network to discover the relevant genes of these two lncRNAs. The FMO6P-related genes were enriched in the Fanconi anemia pathway and cancer transcriptional misregulation. Furthermore, the PRR26-related genes were enriched in Transcriptional misregulation in cancer, the MAPK signaling pathway, in tight-junction Proteoglycans in cancer, and in Protein digestion and absorption, most of which are classical signaling pathways closely related to the genesis and progression of cancer. For example, the MAPK signaling pathway has been proposed to be associated with the occurrence, invasion, and metastasis of LUSC [50]. Therefore, functional enrichment analysis may elucidate the role of FMO6P and PRR26 in carcinogenesis of LUSC.

Although the findings of the present study might have significant clinical implications, several limitations should be considered. First, a longer follow-up time is needed to validate our findings. Second, apart from data from the TCGA database, other experimental methods are required to verify the present findings. Third, the function of FMO6P and PRR26 in LUSC needs to be further studied.

In conclusion, the present study identified a two-lncRNA signature as a potential outcome predictor for LUSC patients via analyzing the genome-wide lncRNA expression profiles from TCGA using a ceRNA network. Future functional investigations are required to explore the mechanisms underlying the roles of these lncRNAs in LUSC.

## MATERIALS AND METHODS

### TCGA dataset and patient information

RNA sequencing (RNA-Seq) data from 504 individuals with LUSC were obtained from the TCGA data portal (https://portal.gdc.cancer.gov/). Exclusion criteria were as follows: 1) histologic diagnosis ruled out LUSC; 2) another malignancy besides LUSC. Overall, 474 LUSC patients were included in this study, including data from 429 LUSC tissue samples and 45 non-tumorous adjacent-normal lung tissue samples up to February 12, 2017. Clinical data, including outcome and staging information on TCGA LUSC patients, were also downloaded from the Data Coordinating Center. Of those 474 patients, there were 159 LUSC patients with lymphatic metastasis and 270 LUSC patients with non-lymphatic metastasis. According to the TNM stage, 343 patients were identified as having well- or moderately differentiated LUSC (stage I-II), and the remaining 86 patients had poorly differentiated LUSC (stage III-IV). Since the data were obtained from the TCGA database, additional approval from the Ethics Committee was not needed. Data processing procedures met the requirements of the data access policies and NIH TCGA human subject protection (http://cancergenome.nih.gov/publications/publicationguidelines).

### Identification of differentially expressed RNAs in LUSC samples

**Figure 11:**
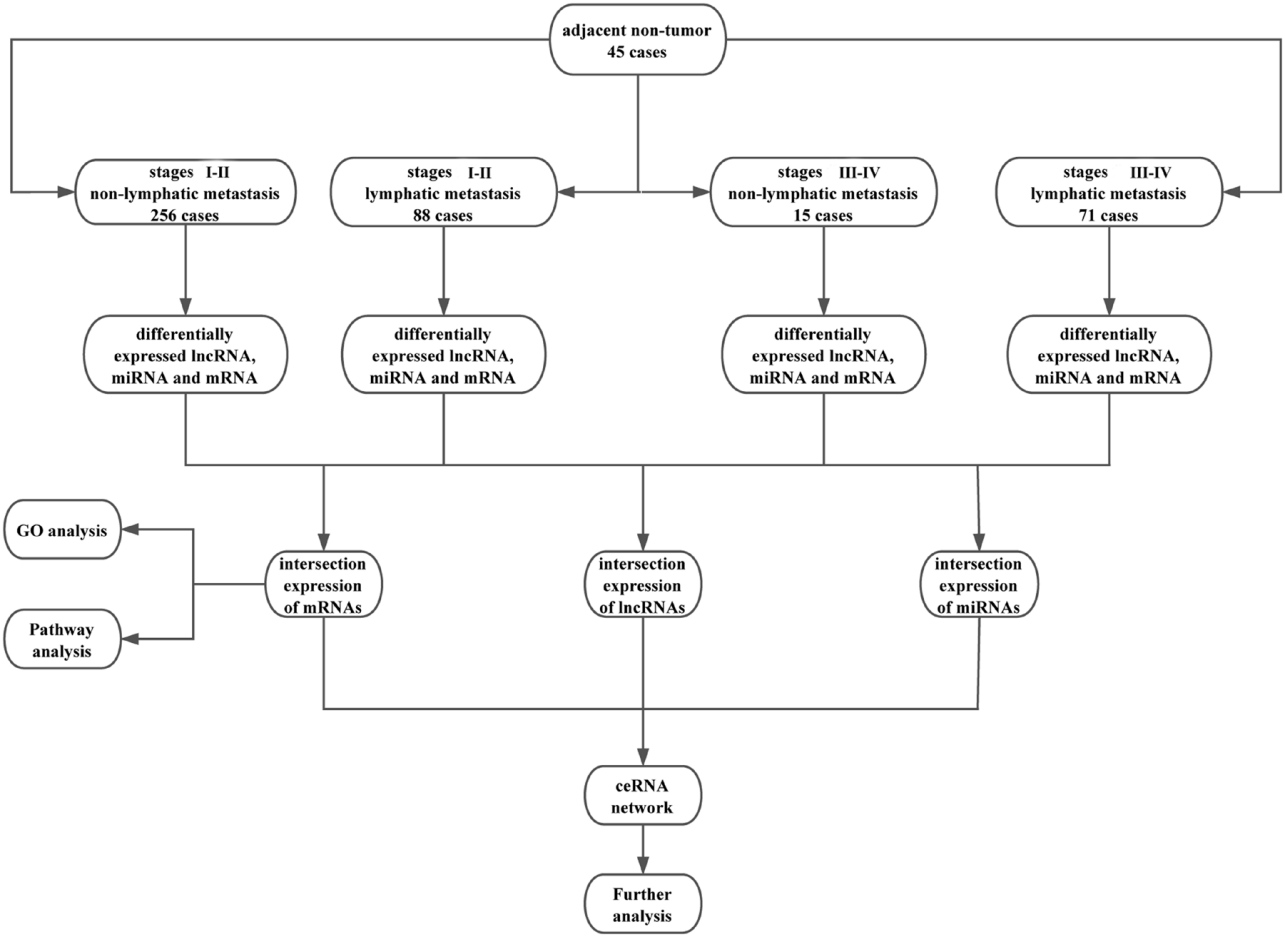
Flow chart of bioinformatics analysis

The expression of human RNA (lncRNA, mRNA, and miRNA) in LUSC samples was analyzed using RNASeqV2 and Illumina HiSeq 2000 miRNA sequencing platforms. Level 3 RNA expression data were collected from TCGA Data Portal and normalized by TCGA [51]. To detect the differential expression of RNA, samples were divided into tumor tissues vs. adjacent non-tumor tissues, stage I-II vs. stage III-IV, and lymphatic metastasis vs. non-lymphatic metastasis, respectively. The flow chart for bioinformatics analysis was given in Figure 11. Since many RNAs were not expressed in particular tissue types or showed little variation among patients in the TCGA database, only RNAs expressed in at least two normal or tumor samples, with at least 100 counts per million were retained in the profile. Expression differences were characterized by fold change and associated P-values. Fold change indicates the difference in expression of each RNA from LUSC to adjacent non-tumor tissues. Up-regulated and down-regulated RNA were assigned fold changes >2 and <0.5, respectively, with FDR-adjusted P<0.05.

### Construction of the ceRNA network and functional assessment

Based on the relationship among lncRNA, mRNA, and miRNA, the ceRNA network was constructed in three steps: i) LUSC-specific RNA (lncRNA, mRNA, and miRNA) filtration: Up-regulated and down-regulated LUSC-specific RNAs were assigned fold changes >2 and <0.5, respectively, with P<0.05. To maximize data reliability, LUSC-specific lncRNAs not registered in GENCODE (http://www.gencodegenes.org/) were abandoned; ii) miRTarBase (http://mirtarbase.mbc.nctu.edu.tw/) and Targetscan (http://www.targetscan.org/) were used to predict the mRNAs targeted by miRNAs; iii) lncRNA-miRNA interactions were predicted using miRanda tools (http://www.microrna.org/microrna/home.do). Furthermore, miRNAs that negatively regulated expression of both lncRNAs and mRNAs were selected for construction of the ceRNA network. Cytoscape v3.0 was used to construct and visualize the ceRNA network [52]. To understand the underlying pathways and biological processes of differentially expressed genes in the ceRNA network, the Database for Annotation, Visualization, and Integrated Discovery (DAVID) (http://david.abcc.ncifcrf.gov/) [53] was employed for functional enrichment analysis, in which KEGG (Kyoto Encyclopedia of Genes and Genomes) pathways and GO (Gene Ontology) biological processes were interested at significance level of P<0.05 and an enrichment score>1.5.

### Construction of the LUSC-specific lncRNA-based prognostic signature

Based on the ceRNA network, LUSC-specific lncRNAs were selected, and the expression level of each lncRNA was log2 transformed for further analysis. The univariate Cox proportional hazards regression model with a significance level set at 0.05 was used to analyze LUSC-specific lncRNAs associated with OS. The prognostic risk score for predicting OS was calculated as previously reported: Risk score=exp_lncRNA1_ *β_lncRNA1_+exp_lncRNA2_*β_lncRNA2_+…exp_lncRNA_*β_lncRNAn_ (exp: expression level, β: the regression coefficient derived from the multivariate Cox regression model) [54]. Utilizing the median risk score as the cutoff point, LUSC patients were divided into high-score and low-score groups [55]. Univariate Cox proportional hazards regression analyses were further conducted to investigate the effects of various clinical characteristics and the risk score on the OS of LUSC patients.

We further analyzed clinical features including gender, tumor stage, TNM staging system, lymphatic metastasis, and patient outcome assessment with lncRNAs in the ceRNA network. The relationship between the LUSC-specific lncRNAs and clinical features was examined using Student’s t-test. Univariate Cox proportional hazards regression analyses were further performed to investigate the effects of various clinical features and risk score on the OS of LUSC. The hazard ratio (HR) and 95% confidence interval (CI) were assessed. HRs for a two-fold change in gene expression from univariate Cox regression analysis were used to identify LUSC-specific lncRNAs associated with OS. lncRNAs defined as having a protective signature showed HR<1 and those defined as high-risk had HR for death>1. A time-dependent receiver operating characteristic (ROC) curve analysis within five years as the defining point was performed with the R package “survival-ROC,” to evaluate the predictive value of the risk score for time-dependent outcomes [56]. Kaplan-Meier and log-rank methods (Mantel-Haenszel test) were used to test the equality of survival distributions in different groups subjected to comparison using IBM SPSS Statistics Version 21 software (SPSS Inc., Chicago, IL, USA). The operating characteristic curve was used to evaluate LUSC-specific lncRNAs for their sensitivity and specificity for detection of LUSC.

## SUPPLEMENTARY MATERIALS TABLES







## Abbreviations

lncRNAs: long non-coding RNAs
ceRNAs: competing endogenous RNAs
LUSC: lung squamous cell carcinoma
OS: overall survival
TCGA: The Cancer Genome Atlas
LUAD: lung adenocarcinoma
TNM: tumor node metastasis
MREs: miRNA response elements
RNA-Seq: RNA sequencing
DAVID: Database for Annotation, Visualization, and Integrated Discovery
KEGG: Kyoto Encyclopedia of Genes and Genomes
GO: Gene Ontology
HR: hazard ratio
CI: confidence interval
ROC: receiver operating characteristic
MIC: maximal information coefficient
AUC: area under ROC curve

## References

[1] Torre LA, Bray F, Siegel RL, Ferlay J, Lortet-Tieulent J, Jemal A (2015). Global cancer statistics, 2012. CA Cancer J Clin.

[2] Chen WJ, Gan TQ, Qin H, Huang SN, Yang LH, Fang YY, Li ZY, Pan LJ, Chen G (2017). Implication of downregulation and prospective pathway signaling of microRNA-375 in lung squamous cell carcinoma. Pathol Res Pract.

[3] Detterbeck FC, Boffa DJ, Tanoue LT (2009). The new lung cancer staging system. Chest.

[4] Kenfield SA, Wei EK, Stampfer MJ, Rosner BA, Colditz GA (2008). Comparison of aspects of smoking among the four histological types of lung cancer. Tob Control.

[5] Mukhopadhyay S, Katzenstein AL (2011). Subclassification of non-small cell lung carcinomas lacking morphologic differentiation on biopsy specimens: utility of an immunohistochemical panel containing TTF-1, napsin A, p63, and CK5/6. Am J Surg Pathol.

[6] Mercer TR, Mattick JS (2013). Structure and function of long noncoding RNAs in epigenetic regulation. Nat Struct Mol Biol.

[7] Cao D, Ding Q, Yu W, Gao M, Wang Y (2016). Long noncoding RNA SPRY4-IT1 promotes malignant development of colorectal cancer by targeting epithelial-mesenchymal transition. Onco Targets Ther.

[8] Li B, Chen P, Qu J, Shi L, Zhuang W, Fu J, Li J, Zhang X, Sun Y, Zhuang W (2014). Activation of LTBP3 gene by a long noncoding RNA (lncRNA) MALAT1 transcript in mesenchymal stem cells from multiple myeloma. J Biol Chem.

[9] Zhou Q, Chen J, Feng J, Wang J (2016). Long noncoding RNA PVT1 modulates thyroid cancer cell proliferation by recruiting EZH2 and regulating thyroid-stimulating hormone receptor (TSHR). Tumour Biol.

[10] Wang SH, Zhang MD, Wu XC, Weng MZ, Zhou D, Quan ZW (2016). Overexpression of LncRNA-ROR predicts a poor outcome in gallbladder cancer patients and promotes the tumor cells proliferation, migration, and invasion. Tumour Biol.

[11] Zheng X, Hu H, Li S (2016). High expression of lncRNA PVT1 promotes invasion by inducing epithelial-to-mesenchymal transition in esophageal cancer. Oncol Lett.

[12] Pickard MR, Williams GT (2016). The hormone response element mimic sequence of GAS5 lncRNA is sufficient to induce apoptosis in breast cancer cells. Oncotarget.

[13] Cheng Z, Bai Y, Wang P, Wu Z, Zhou L, Zhong M, Jin Q, Zhao J, Mao H, Mao H (2017). Identification of long noncoding RNAs for the detection of early stage lung squamous cell carcinoma by microarray analysis. Oncotarget.

[14] Liu B, Chen Y, Yang J (2017). LncRNAs are altered in lung squamous cell carcinoma and lung adenocarcinoma. Oncotarget.

[15] Zhang S, Zhong G, He W, Yu H, Huang J, Lin T (2016). lncRNA up-regulated in nonmuscle invasive bladder cancer facilitates tumor growth and acts as a negative prognostic factor of recurrence. J Urol.

[16] Zhang J, Zhang B, Wang T, Wang H (2015). LncRNA MALAT1 overexpression is an unfavorable prognostic factor in human cancer: evidence from a meta-analysis. Int J Clin Exp Med.

[17] Zhou M, Zhao H, Wang Z, Cheng L, Yang L, Shi H, Yang H, Sun J (2015). Identification and validation of potential prognostic lncRNA biomarkers for predicting survival in patients with multiple myeloma. J Exp Clin Cancer Res.

[18] Yang F, Lv SX, Lv L, Liu YH, Dong SY, Yao ZH, Dai XX, Zhang XH, Wang OC (2016). Identification of lncRNA FAM83H-AS1 as a novel prognostic marker in luminal subtype breast cancer. Onco Targets Ther.

[19] Huang GQ, Ke ZP, Hu HB, Gu B (2017). Co-expression network analysis of long noncoding RNAs (IncRNAs) and cancer genes revealsSFTA1P and CASC2abnormalities in lung squamous cell carcinoma. Cancer Biol Ther.

[20] Salmena L, Poliseno L, Tay Y, Kats L, Pandolfi PP (2011). A ceRNA hypothesis: the Rosetta Stone of a hidden RNA language?. Cell.

[21] Zhang Y, Xu Y, Feng L, Li F, Sun Z, Wu T, Shi X, Li J, Li X (2016). Comprehensive characterization of lncRNA-mRNA related ceRNA network across 12 major cancers. Oncotarget.

[22] Zhang J, Fan D, Jian Z, Chen GG, Lai PB (2015). Cancer specific long noncoding RNAs show differential expression patterns and competing endogenous RNA potential in hepatocellular carcinoma. PLoS One.

[23] Zhu Q, Lv T, Wu Y, Shi X, Liu H, Song Y (2017). Long non-coding RNA 00312 regulated by HOXA5 inhibits tumour proliferation and promotes apoptosis in non-small cell lung cancer. J Cell Mol Med.

[24] Yu L, Fang F, Lu S, Li X, Yang Y, Wang Z (2017). lncRNA-HIT promotes cell proliferation of non-small cell lung cancer by association with E2F1. Cancer Gene Ther.

[25] Wang G, Chen H, Liu J (2015). The long noncoding RNA LINC01207 promotes proliferation of lung adenocarcinoma. Am J Cancer Res.

[26] Wang M, Dong X, Feng Y, Sun H, Shan N, Lu T (2017). Prognostic role of the long non-coding RNA, SPRY4 Intronic Transcript 1, in patients with cancer: a meta-analysis. Oncotarget.

[27] Riveiro ME, Astorgues-Xerri L, Vazquez R, Frapolli R, Kwee I, Rinaldi A, Odore E, Rezai K, Bekradda M, Inghirami G, D'Incalci M, Noel K, Cvitkovic E (2016). OTX015 (MK-8628), a novel BET inhibitor, exhibits antitumor activity in non-small cell and small cell lung cancer models harboring different oncogenic mutations. Oncotarget.

[28] Wang Y, Yang Y, Zhu Y, Li L, Chen F, Zhang L (2017). Polymorphisms and expression of IL-32: impact on genetic susceptibility and clinical outcome of lung cancer. Biomarkers.

[29] Jiang W, Zhang W, Wu L, Liu L, Men Y, Wang J, Liang J, Hui Z, Zhou Z, Bi N, Wang L (2017). MicroRNA-related polymorphisms in PI3K/Akt/mTOR pathway genes are predictive of limited-disease small cell lung cancer treatment outcomes. Biomed Res Int.

[30] Lin K, Xu T, He BS, Pan YQ, Sun HL, Peng HX, Hu XX, Wang SK (2016). MicroRNA expression profiles predict progression and clinical outcome in lung adenocarcinoma. Onco Targets Ther.

[31] Zhu SP, Wang JY, Wang XG, Zhao JP (2017). Long intergenic non-protein coding RNA 00858 functions as a competing endogenous RNA for miR-422a to facilitate the cell growth in non-small cell lung cancer. Aging (Albany NY).

[32] Xue Y, Ni T, Jiang Y, Li Y (2017). LncRNA GAS5 inhibits tumorigenesis and enhances radiosensitivity by suppressing miR-135b expression in non-small cell lung cancer. Oncol Res.

[33] Cheng DL, Xiang YY, Ji LJ, Lu XJ (2015). Competing endogenous RNA interplay in cancer: mechanism, methodology, and perspectives. Tumour Biol.

[34] Sui J, Li YH, Zhang YQ, Li CY, Shen X, Yao WZ, Peng H, Hong WW, Yin LH, Pu YP, Liang GY (2016). Integrated analysis of long non-coding RNAassociated ceRNA network reveals potential lncRNA biomarkers in human lung adenocarcinoma. Int J Oncol.

[35] Yan Y, Fan Q, Wang L, Zhou Y, Li J, Zhou K (2017). LncRNA Snhg1, a non-degradable sponge for miR-338, promotes expression of proto-oncogene CST3 in primary esophageal cancer cells. Oncotarget.

[36] Wang Y, Yang T, Zhang Z, Lu M, Zhao W, Zeng X, Zhang W (2017). Long non-coding RNA TUG1 promotes migration and invasion by acting as a ceRNA of miR-335-5p in osteosarcoma cells. Cancer Sci.

[37] Deng J, Liang Y, Liu C, He S, Wang S (2015). The up-regulation of long non-coding RNA AFAP1-AS1 is associated with the poor prognosis of NSCLC patients. Biomed Pharmacother.

[38] Park S, Shimizu C, Shimoyama T, Takeda M, Ando M, Kohno T, Katsumata N, Kang YK, Nishio K, Fujiwara Y (2006). Gene expression profiling of ATP-binding cassette (ABC) transporters as a predictor of the pathologic response to neoadjuvant chemotherapy in breast cancer patients. Breast Cancer Res Treat.

[39] Ozes AR, Wang Y, Zong X, Fang F, Pilrose J, Nephew KP (2017). Therapeutic targeting using tumor specific peptides inhibits long non-coding RNA HOTAIR activity in ovarian and breast cancer. Sci Rep.

[40] Shen Y, Wang Z, Loo LW, Ni Y, Jia W, Fei P, Risch HA, Katsaros D, Yu H (2015). LINC00472 expression is regulated by promoter methylation and associated with disease-free survival in patients with grade 2 breast cancer. Breast Cancer Res Treat.

[41] Yue B, Sun B, Liu C, Zhao S, Zhang D, Yu F, Yan D (2015). Long non-coding RNA Fer-1-like protein 4 suppresses oncogenesis and exhibits prognostic value by associating with miR-106a-5p in colon cancer. Cancer Sci.

[42] Zheng S, Zheng D, Dong C, Jiang J, Xie J, Sun Y, Chen H Development of a novel prognostic signature of long non-coding RNAs in lung adenocarcinoma. J Cancer Res Clin Oncol 2017.

[43] Song P, Jiang B, Liu Z, Ding J, Liu S, Guan W (2017). A three-lncRNA expression signature associated with the prognosis of gastric cancer patients. Cancer Med.

[44] Droop J, Szarvas T, Schulz WA, Niedworok C, Niegisch G, Scheckenbach K, Hoffmann MJ (2017). Diagnostic and prognostic value of long noncoding RNAs as biomarkers in urothelial carcinoma. PLoS One.

[45] Xie F, Xiang X, Huang Q, Ran P, Yuan Y, Li Q, Qi G, Guo X, Xiao C, Zheng S (2017). Reciprocal control of lncRNA-BCAT1 and beta-catenin pathway reveals lncRNA-BCAT1 long non-coding RNA acts as a tumor suppressor in colorectal cancer. Oncotarget.

[46] Lv L, Wei M, Lin P, Chen Z, Gong P, Quan Z, Tang Z (2017). Integrated mRNA and lncRNA expression profiling for exploring metastatic biomarkers of human intrahepatic cholangiocarcinoma. Am J Cancer Res.

[47] Nie ZL, Wang YS, Mei YP, Lin X, Zhang GX, Sun HL, Wang YL, Xia YX, Wang SK Prognostic significance of long noncoding RNA Z38 as a candidate biomarker in breast cancer. J Clin Lab Anal 2017.

[48] Gao X, Wu Y, Yu W, Li H (2016). Identification of a seven-miRNA signature as prognostic biomarker for lung squamous cell carcinoma. Oncotarget.

[49] Hu XG, Chen L, Wang QL, Zhao XL, Tan J, Cui YH, Liu XD, Zhang X, Bian XW (2016). Elevated expression of ASCL2 is an independent prognostic indicator in lung squamous cell carcinoma. J Clin Pathol.

[50] Yu GP, Chen GQ, Wu S, Shen K, Ji Y (2011). The expression of PEBP4 protein in lung squamous cell carcinoma. Tumour Biol.

[51] Zhao Y, Simon R (2008). BRB-ArrayTools Data Archive for human cancer gene expression: a unique and efficient data sharing resource. Cancer Inform.

[52] Shannon P, Markiel A, Ozier O, Baliga NS, Wang JT, Ramage D, Amin N, Schwikowski B, Ideker T (2003). Cytoscape: a software environment for integrated models of biomolecular interaction networks. Genome Res.

[53] Qiu M, Feng D, Zhang H, Xia W, Xu Y, Wang J, Dong G, Zhang Y, Yin R, Xu L (2016). Comprehensive analysis of lncRNA expression profiles and identification of functional lncRNAs in lung adenocarcinoma. Oncotarget.

[54] Zeng JH, Liang L, He RQ, Tang RX, Cai XY, Chen JQ, Luo DZ, Chen G (2017). Comprehensive investigation of a novel differentially expressed lncRNA expression profile signature to assess the survival of patients with colorectal adenocarcinoma. Oncotarget.

[55] Zhou X, Huang Z, Xu L, Zhu M, Zhang L, Zhang H, Wang X, Li H, Zhu W, Shu Y, Liu P (2016). A panel of 13-miRNA signature as a potential biomarker for predicting survival in pancreatic cancer. Oncotarget.

[56] Heagerty PJ, Lumley T, Pepe MS (2000). Time-dependent ROC curves for censored survival data and a diagnostic marker. Biometrics.

