# Supplementary material for: Integrated analysis of competing endogenous RNA network revealing lncRNAs as potential prognostic biomarkers in human lung squamous cell carcinoma
Source: Oncotarget. 2017 Jul 27;8(39):65997–6018. doi: 10.18632/oncotarget.19627 (PMC5630388; doi:10.18632/oncotarget.19627)
Supplement: Supplementary file 1 [file oncotarget-08-65997-s001.pdf]

Integrated analysis of competing endogenous RNA network revealing lncRNAs as potential prognostic biomarkers in human lung squamous cell carcinoma

SUPPLEMENTARY MATERIALS

Supplementary Table 1: Dysregulated lncRNA in LUSC

Regulated style	lncRNA
**Down**	CYP2B7P, GGT3P, GGTA1P, HLA-DRB6, MEIS3P1, TRPC2, CMAHP, MBL1P, KIAA0087, LINC01558, LINC00312, LOC100499484-C9ORF174, LINC00341, LINC00472, AGPAT4-IT1, TUBA4B, MIR22HG, LOC90246, AOC4P, PSMG3-AS1, ZNF300P1, LINC00261, LINC00924, MGC27382, CHIAP2, ABCC13, UMODL1-AS1, LINC01105, WWC2-AS2, LINC00689, LINC00961, CYP4Z2P, ABHD11-AS1, DNM1P46, SFTA1P, NAPSB, TRHDE-AS1, LINC00482, LINC00908, SIGLEC17P, LOC284578, CYP1B1-AS1, FLJ34503, KRTAP5-AS1, LINC01140, PWARSN, CCL15-CCL14, PICSAR, TPTEP1, GVINP1, MIR99AHG, RPL13AP17, SIGLEC16, FLJ26850, C1orf140, WDFY3-AS2, PRR26, LINC00982, C5orf56, LOC441204, PGM5-AS1, PGM5P2, BCRP3, ABCC6P1, NCF1B, NCF1C, LHFPL3-AS2, ABCC6P2, SNORD116-4, LINC00930, LINC00092, BRE-AS1
**Up**	ALOX12P2, CYP2D7, PVT1, SPRR2C, AURKAPS1, SNHG1, PART1, DGCR5, PTTG3P, OR7E91P, FER1L4, AFAP1-AS1, LOC93429, TCAM1P, LOC148709, MSL3P1, HOXA11-AS, MIR9-3HG, LINC01559, LINC00319, LOC285629, DLX6-AS1, FIRRE, LINC00634, SOX2-OT, TPRXL, SLC6A10P, FMO6P, LOC399815, C1orf220, DDX12P, LOC440173, CDIPT-AS1, FAR2P1, GEMIN8P4, MIR31HG, DUSP5P1, KC6, LOC642846, LINC00520, RAET1K, MIR924HG, UCA1, SCARNA12, SNORD1C, SNHG4, LOC728554, DIRC3, HOTAIR, FAM86JP, TMPO-AS1, KTN1-AS1, LINC00887, LINC00704, LINC00173

Supplementary Table 2: Dysregulated miRNA in LUSC

Regulated style	miRNA	Sequence
Down	hsa-miR-30a-5p	UGUAAACAUCCUCGACUGGAAG
	hsa-miR-30a-3p	CUUUCAGUCGGAUGUUUGCAGC
	hsa-miR-101-5p	CAGUUAUCACAGUGCUGAUGCU
	hsa-miR-30c-2-3p	CUGGGAGAAGGCUGUUUACUCU
	hsa-miR-30d-5p	UGUAAACAUCCCCGACUGGAAG
	hsa-miR-30d-3p	CUUUCAGUCAGAUGUUUGCUGC
	hsa-miR-139-5p	UCUACAGUGCACGUGUCUCCAGU
	hsa-miR-139-3p	UGGAGACGCGGCCCUGUUGGAGU
	hsa-miR-218-5p	UUGUGCUUGAUCUAACCAUGU
	hsa-miR-133a-3p	UUUGGUCCCCUUCAACCAGCUG
	hsa-miR-144-5p	GGAUAUCAUCAUAUACUGUAAG
	hsa-miR-144-3p	UACAGUAUAGAUGAUGUACU
	hsa-miR-126-5p	CAUUAUUACUUUUGGUACGCG
	hsa-miR-190a-5p	UGAUAUGUUUGAUAUAUUAGGU
	hsa-miR-375	UUUGUUCGUUCGGCUCGCGUGA
	hsa-miR-326	CCUCUGGGCCCUUCCUCCAG
	hsa-miR-338-5p	AACAAUAUCCUGGUGCUGAGUG
	hsa-miR-338-3p	UCCAGCAUCAGUGAUUUUGUUG
	hsa-miR-133b	UUUGGUCCCCUUCAACCAGCUA
	hsa-miR-451a	AAACCGUUACCAUUACUGAGUU
	hsa-miR-486-5p	UCCUGUACUGAGCUGCCCCGAG
	hsa-miR-490-3p	CAACCUGGAGGACUCCAUGCUG
	hsa-miR-511-5p	GUGUCUUUUGCUCUGCAGUCA
	hsa-miR-497-5p	CAGCAGCACACUGUGGUUUGU
	hsa-miR-3065-3p	UCAGCACCAGGAUAUUGUUGGAG
Up	hsa-miR-31-5p	AGGCAAGAUGCUGGCAUAGCU
	hsa-miR-31-3p	UGCUAUGCCAACAUAUUGCCAU
	hsa-miR-96-5p	UUUGGCACUAGCACAUUUUUGCU
	hsa-miR-196a-5p	UAGGUAGUUUCAUGUUGUUGGG
	hsa-miR-182-5p	UUUGGCAAUGGUAGAACUCACACU
	hsa-miR-183-5p	UAUGGCACUGGUAGAAUUCACU
	hsa-miR-205-5p	UCCUUCAUUCCACCGGAGUCUG
	hsa-miR-210-3p	CUGUGCGUGUGACAGCGGCUGA
	hsa-miR-9-5p	UCUUUGGUUAUCUAGCUGUAUGA
	hsa-miR-130b-5p	ACUCUUUCCCUGUUGCACUAC
	hsa-miR-130b-3p	CAGUGCAAUGAUGAAAGGGCAU
	hsa-miR-196b-5p	UAGGUAGUUUCCUGUUGUUGGG
	hsa-miR-629-3p	GUUCUCCCAACGUAAGCCCAGC
	hsa-miR-708-5p	AAGGAGCUUACAAUCUAGCUGGG
	hsa-miR-708-3p	CAACUAGACUGUGAGCUUCUAG
	hsa-miR-944	AAAUUAUUGUACAUCGGAUGAG
	hsa-miR-1269a	CUGGACUGAGCCGUGCUACUGG

Supplementary Table 3: Dysregulated mRNA in LUSC

See Supplementary File 1

Supplementary Table 4: Dysregulated KEGG pathway and GO term in LUSC

See Supplementary File 2
