# Supplementary material for: Integrated analysis of competing endogenous RNA network revealing lncRNAs as potential prognostic biomarkers in human lung squamous cell carcinoma
Source: Oncotarget. 2017 Jul 27;8(39):65997–6018. doi: 10.18632/oncotarget.19627 (PMC5630388; doi:10.18632/oncotarget.19627)
Supplement: Supplementary file 2 [file oncotarget-08-65997-s002.docx]

**Supplementary Table 3: Dysregulated mRNA in LUSC**

| **Regulated style** | **mRNA** |
| --- | --- |
| **Down** | A2M， ABCA3， ACACB， ACADL， ACP5， ACTG2， ACTN2， ACVRL1， ADARB1， ADCY8， ADCY9， ADH1A， ADH1B， ADPRH， ADRA1B， ADRA1A， ADRB1， ADRB2， AGER， AGRP， AGTR1， AGTR2， AK1， ALAS2， ALDH2， ALDH3B1， ALOX5， ALOX5AP， ALOX15B， ALPL， ALPP， ALPPL2， AMBP， AMY1A， ANG， ANGPT1， ANK2， ANPEP， ANXA3， AOX1， APBB1， APOA1， APOC1， APOH， AQP1， AQP4， AQP5， AQP7， AQP9， AR， ABCC6， RHOB， ARHGAP6， ARRB1， ARSE， ART4， ASAH1， ASPA， ATF3， ATP1A2， ATP1B2， AVPR2， AZGP1， AZU1， ADGRB3， BMP2， BMP3， BMP5， BMX， BST1， KLF9， BTK， SERPING1， C1QA， C1QB， C2， C3， C4A， C4BPA， C4BPB， C5， C5AR1， C6， C7， C8B， MYRF， CA3， CA4， CACNA1C， CACNA1D， CACNA1S， CACNB4， CAMK2A， CAMP， CAPN3， CASP5， CASQ2， CAT， CAV1， CAV2， CAV3， RUNX1T1， CCIN， CD1C， CD1E， CD4， CD5L， CD22， CD33， SIGLEC6， CD34， CD36， CD37， CD40LG， CD53， CD68， CD69， CD74， ADGRE5， CDH5， CDO1， CDR1， CD52， CETP， CFL2， CFTR， CEACAM3， CEACAM8， CEACAM4， CHI3L2， CHIT1， CHRM1， CHRM2， CHRNA2， CISH， CKMT2， CLIC2， CMA1， CCR1， CNGA4， CNN1， CNR1， CNTFR， COL4A3， COL4A4， COL13A1， KLF6， COX7A1， CPA3， CPB2， CPM， CR1， CRIP1， CRY2， CRYM， CSF1， CSF2， CSF2RA， CSF3， CSF3R， CST5， CST6， CTGF， CTNND2， CTSE， CTSG， CTSH， CTSS， CTSW， CX3CR1， CYB5A， CYBB， CYP1A2， CYP2A6， CYP3A7， CYP2F1， CYP3A5， CYP4B1， CYP17A1， CYP21A2， CYP27A1， DAB2， DACH1， CD55， DAPK1， DCC， DCN， ACE， DDC， DES， CFD， DMBT1， DNAH6， DNAH9， DNASE1L3， DOCK2， DPP4， DPP6， DPT， DPYS， DPYSL2， DRD1， TSC22D3， HBEGF， ECM2， DUSP1， DUSP8， S1PR1， EDN1， EDN3， EDNRB， EGR1， ELANE， ELF5， ELN， EMP2， ADGRE1， ENG， EPAS1， ERBB4， ERG， ETS1， ETV1， EVI2B， F8， F10， F11， FABP4， FABP3， EFEMP1， FBP1， FCER1A， MS4A2， FCGR3A， FCGR3B， FCN1， FGA， FGF2， FGF10， FGF14， GPC5， FGFR4， FGG， FGR， FHL1， FIGF， FOXF1， FOXJ1， FLI1， FLNC， FLT3， FLT4， FMO2， FMO5， FOLR1， FOLR3， FOS， FOSB， FPR1， FPR2， NR5A2， ACKR1， GABRB2， LRRC32， GAS6， GATA1， GATA2， GATA6， GDF10， GFRA1， GFRA2， GJA4， GJA5， GJB1， GLRX， GNG7， GNG11， GP9， GPD1， GPM6A， GPR4， GPR17， GPR20， GPER1， GPR39， GRK5， GPX3， GRIA1， GRID1， GRIK4， CXCL2， CXCL3， GSTA3， GSTM5， GUCY1A2， GUCA2A， GYPC， GYPE， HBA1， HBA2， HBB， HBG1， HBG2， SERPIND1， HDC， NCKAP1L， HGF， HK3， HLA-DMA， HLA-DOA， HLA-DPA1， HLA-DPB1， HLA-DQA1， HLA-DQA2， HLA-DQB1， HLA-DQB2， HLA-DRA， HLA-DRB1， HLA-DRB5， HLF， HMGCS2， NR4A1， FOXA2， HOXA5， HPCAL1， HPD， HPGD， HPN， HPR， HRC， HSPB2， HYAL1， ICAM1， ICAM2， ICAM4， IRF8， ID4， CFI， IGFALS， CYR61， IHH， IL3RA， IL5RA， IL6R， IL6ST， IL7R， CXCR1， CXCR2， ITGA1， ITGA9， ITGAL， ITGAM， ITGAX， ITGB2， ITIH2， ITIH3， ITPR1， JUND， ANOS1， KCNA3， KCNA4， KCNA5， KCNB1， KCNE1， KCNJ5， KCNJ15， KCNK3， KCNQ1， KDR， KIR2DL1， KIR2DL3， KIR2DS4， KIR3DL1， KIR3DL2， KIT， KLRB1， KLRD1， KRT4， KRT7， AFF3， LAIR1， LAMA2， LAMB2， LCP1， LDLR， LEPR， LGALS4， LIFR， LMO2， LMO7， LPL， LRP2， LSAMP， LTA4H， LTC4S， LTK， LYZ， SMAD6， SMAD9， MAG， MAK， MAL， MAOA， MAOB， MAP6， MATN3， MEF2C， MEFV， MEOX2， MFAP4， MFNG， SCGB2A1， MGAT3， MGP， CIITA， MITF， NR3C2， MME， MMP19， MNDA， MRC1， MSR1， MSRA， MT1A， MT1M， MYO1F， MUC1， MUSK， GADD45B， MYH1， MYH2， MYH10， MYH11， MYL3， MYO1A， MYO7B， MYOC， CEACAM6， NCF2， NEDD9， NFATC2， NFE2， NINJ2， NKG7， NOTCH4， NOVA2， NPR1， NPY1R， NRGN， NTRK3， ROR1， DDR2， NR4A2， OGN， OLR1， ORM1， OTC， P2RX1， P2RX7， PCDH1， PCDH9， PCSK2， PDE1C， PDE2A， PDE4C， PDE4D， PDE1B， PDK4， PECAM1， PEG3， PER1， PF4， CFP， PGC， PGM5， PGR， ABCB1， SERPINA1， PIGR， PIK3CG， PLA2G1B， PLA2G5， PLCB2， PLCL1， FXYD1， PNMT， UBL3， SEPT4， PODXL， PPARG， PPBP， NPY4R， PRELP， PRF1， SRGN， PRKCB， PRKCE， PRKCQ， PRKG1， MAPK4， PROS1， MASP1， RELN， PTAFR， PTGDS， PTGFR， PTGIR， PTGIS， PTH1R， PTPRB， PTPRC， PTPRM， PTPRN2， PTPRO， PYGM， PZP， RAB27A， RARA， RARRES3， RASGRF1， RBMS2， RBP2， RBP4， REN， RFX2， RGS13， RNASE1， RNASE4， ROBO2， RORB， RORC， ROS1， RP1， RPS6KA2， RRAD， RRAS， RS1， RTN1， RXRG， RYR2， SORT1， S100A4， ACSM3， SCN1A， SCN2B， SCN4B， SCN7A， SCNN1B， SCNN1G， SCTR， SRL， CCL2， CCL14， CCL15， CCL17， CCL21， CCL23， CCL24， CXCL5， CXCL12， SELE， SELP， SELPLG， SEPP1， SFRP5， SFTPB， SFTPC， SFTPD， SGCA， SGCG， SH3GL2， SH3GL3， SHH， SLA， SLC1A1， SLC1A7， SLC4A1， SLC5A1， SLC5A4， SLC6A4， SLC6A12， SLC6A13， SLC7A2， SLC8A3， SLC10A2， SLC11A1， SLC14A1， SLC15A2， SLC18A2， SLCO2A1， SLC22A3， SLIT3， SLPI， SIGLEC1， SOD3， SPI1， SPN， SPTBN1， SRD5A2， SSTR1， STAC， SULT1A2， SULT1A1， TACC1， TAL1， TBX2， TBX5， TBXAS1， HNF1B， ZEB1， TCF21， TEK， NR2F1， TFPI， LEFTY2， TGFBR2， TGFBR3， TGM2， THBD， TIE1， TIMP3， NKX2-1， TLE2， TLL1， TLR3， TLR4， TSPAN7， TSPAN8， TM4SF4， TSPAN4， TMOD1， TMPRSS2， CLDN5， CLEC3B， TNNC1， TNNI2， TNR， TNS1， TNXB， TPSAB1， TRPC3， TRPC6， TXK， TYROBP， SCGB1A1， UGT2B4， COL14A1， UTRN， VIP， VIPR1， VTN， VWF， LAT2， WFS1， WNT2， WNT7A， XPNPEP2， ZFP36， ZBTB16， DNALI1， LAPTM5， MALL， SEMA3B， KCNAB1， LST1， NR4A3， GDF5， SYN3， DYSF， ACOX2， AXIN2， FZD4， GFI1B， NME5， PIP5K1B， PLA2G10， SPARCL1， SRPX， LTBP4， NR0B2， RECK， SDPR， SORBS2， GPR65， RGS5， ITGA10， ITGA8， GAS7， DDO， FCN3， KMO， SCARF1， PDE8B， HSD17B6， OASL， AOC3， DCHS1， PDE5A， SLC4A4， STX11， MARCO， HYAL2， S1PR4， B3GALT2， MYOM1， TNFSF14， TNFSF13， TNFSF12， SIGLEC5， RGS9， TNFRSF10C， SCEL， CDKL1， FGF18， LIN7A， NRP1， WISP2， ALDH1A2， APLN， ST3GAL5， VNN2， CACNA1H， MGAM， SELENBP1， PGLYRP1， LIMD1， CDKL2， F2RL3， CLIC3， CCRL2， SEMA5A， DOK2， GPRC5A， SLC7A7， PAPSS2， ANGPTL1， CLDN2， DIRAS3， LDB2， RGN， SLC16A5， SLC16A4， IL1RL1， REPS2， MAGI1， CACNA2D2， CD83， CD163， SLC9A3R2， SLIT2， KL， CD101， ARHGAP29， FAM189A2， ABCG2， NCR1， LY86， ITM2A， FHL5， ARHGEF6， SH3BP5， THEMIS2， NAPSA， TBX4， XAGE2， ADAMTS1， GMFG， NRG2， SPAG6， AKAP12， AATK， GNA14， ARHGEF10， LPIN2， KIAA0040， RAPGEF2， GPRIN2， SEMA3E， SECISBP2L， KIAA0408， DOCK4， PHACTR2， FAM65B， STARD8， RASSF2， SPOCK2， ZEB2， GAB2， ARHGAP44， KBTBD11， P2RY14， CD302， DLEC1， SH2D3C， ABCC9， TSPAN32， ENAM， FRY， LHFP， CALCRL， PRG4， ANGPTL7， GPA33， RCAN2， MSLN， RASGRP2， IGSF6， RAMP2， RAMP3， FSTL3， NMUR1， LAMC3， KLHL41， SIRPB1， CLEC4M， ABCA9， ABCA8， KLF2， CITED2， CORO2B， DLC1， ATP8A1， MYL9， RAPGEF3， CLEC10A， UNC13B， FBLN5， NEBL， SLC34A2， SORBS1， IFITM2， COLEC10， TXNIP， CELF2， PNMA2， NRG3， RAI2， CAPN9， NES， IQGAP2， CYSLTR1， WASF3， FAXDC2， HCST， CD300C， PPARGC1A， MMP24， LYVE1， APOBEC2， ADIRF， FERMT2， LILRB5， KLK11， LILRA1， LILRB3， LILRA2， RBPMS， C10orf10， RAPGEF4， TPPP， ADAM29， ADAMTS8， PTPN21， PKIG， PLA2G16， HHLA2， LDB3， FAM107A， INMT， WIF1， IRAK3， AKAP13， AKAP2， MST1L， NXPH3， PTGDR2， SLC6A14， FILIP1L， SLCO2B1， GPR182， VSIG4， MGLL， SYNPO， MRAS， NTNG1， CNKSR2， ENPP4， ABLIM3， DENND3， ARHGEF15， MMRN1， CD93， P2RX2， EFR3B， MYH15， LIMCH1， SAMD4A， PDZD2， ZNF423， GPD1L， RGL1， RFTN1， MLC1， RHOBTB2， ARC， COBL， ATP11A， ADGRL2， WWC1， KIF13B， SASH1， SYNM， SYNE1， FAM189A1， TNS2， AHCYL2， ZFPM2， ABCA6， CBX7， DAAM2， PIK3R5， TSPAN12， PADI4， DDAH1， VSIG2， DAPK2， PPP1R15A， CADM1， FLRT3， CFAP45， LMOD1， METTL7A， PARM1， FAM149A， SPEF1， ABI3BP， TRIM58， MYRIP， CNRIP1， SOSTDC1， SAMHD1， KANK2， SNED1， PCDHGA12， PPP1R16B， DNM3， CFAP61， ARHGEF26， CCDC69， RGS22， SPAG8， CLEC4E， HSPB8， LATS2， PCOLCE2， CYFIP2， TPK1， DNAI1， ANKRD1， LAMP3， TSPAN13， CACNG4， CLUL1， DKK2， HSPB7， TJP3， PALD1， FILIP1， DENND2A， CPAMD8， CHIA， GNMT， SULT1C4， PCDH17， CNTN6， SULT1B1， TEKT2， RND1， RBMS3， HPGDS， TOX3， CDH19， SLC27A6， RGCC， KLF15， STXBP6， C11orf21， PDZRN4， TRHDE， PILRA， LMCD1， TBX21， KCNIP1， EHD2， DHH， CELA2B， TNNI3K， PLLP， EGFL7， HSD17B11， PLCE1， CLDN18， CLEC1A， TLR7， RASL12， PCDH12， SLC15A3， TLR8， PLAC8， MS4A4A， KLRF1， ZMYND10， PLA1A， ANGPT4， TRPV2， BIN2， DCDC2， ACKR4， TPPP3， ACSL5， EMCN， UPB1， HIGD1B， IL17D， SHC3， CLIC5， FXYD6， FXYD4， P2RY13， DUOX1， TREM1， SOX18， CYTL1， FAM105A， ADAMTSL4， RHOF， HMGCLL1， APBB1IP， ROBO4， DLL4， L1TD1， LRRN3， SLC6A20， EPDR1， HYDIN， GIPC2， ARHGEF38， PAQR5， SNRK， RASIP1， RNF125， ODAM， ACSM5， MARC2， PID1， BANK1， SPATA6L， IFT57， CRTAC1， ARMC4， SLC47A1， CASC1， TMEM100， LRRC36， ACOXL， GIMAP4， SPTLC3， DRAM1， GIMAP5， VNN3， HEMGN， CAMK2N1， ELMOD1， STAB2， ITLN1， PPP1R9A， SYBU， FLVCR2， LIMS2， RADIL， FAR2， LRP2BP， PAG1， LMO3， THSD1， APOBR， MYO5C， NXF3， PCDHGB7， PCDHGB6， PCDHGA9， PCDHGA6， PCDHGA5， PCDHGA3， PCDHGA2， PCDHAC2， PCDHAC1， PCDHA13， PCDHA12， PCDHA11， PCDHA10， PCDHA3， TEX14， DNAH7， LMOD3， SUSD2， CRTAM， PRMT8， C11orf16， RETN， C8orf4， ANKS1B， SEMA3G， LHX9， C14orf132， PLSCR4， CASS4， ANO2， EPPIN， RGL3， JPH2， ADAMTSL3， ATP10A， ADGRG6， ARFGEF3， RHOJ， GALNT16， DSCAML1， SHROOM4， HEG1， ZNF608， CASKIN2， ARHGAP31， HECW2， KCTD16， CGN， SORCS2， ALPK3， CEP126， ARHGAP20， PCDH10， KIF17， PREX1， FNIP2， KIAA1456， KIAA1462， SHROOM3， NYAP2， LRCH2， EPB41L5， WDFY4， PRX， GBA3， CCDC181， CADM3， SLC46A2， WFDC1， CXCL16， PLEKHB1， MS4A7， RHOU， NLRC4， JAM2， DNASE2B， MYOZ1， NTN4， CACNG6， RXFP1， CCDC81， HPSE2， PKNOX2， PIEZO2， PRDM16， PARVG， C10orf54， SLC39A8， ADGRL4， NECAB1， DPEP2， PDLIM2， RAB17， SOX17， HIF3A， GREM2， HHIP， WBSCR17， DNAI2， TPSB2， TEKT3， CSRNP1， TMPRSS3， TNS3， P2RY12， PCDH20， REEP1， CDK15， PCDH15， STK33， TMEM108， DUSP26， MLPH， C1orf116， MMP28， LILRA6， PRR15L， IRX1， MMEL1， LRRC2， RNF128， RIC3， ACSS3， NDNF， TNFAIP8L2， FAM184A， FAT4， TMEM204， VEPH1， STEAP4， ZBBX， CXorf36， ADGB， C1orf115， LRRC31， RERGL， MMRN2， WDR78， EFCC1， PEAK1， LONRF3， TMC5， FAM124B， LPCAT1， CYBRD1， BTNL8， C2orf54， FAM110D， SYNPO2L， C10orf95， PLPPR3， CPED1， SVEP1， WWC2， ZFP2， MAP3K19， CCDC33， CCDC170， MROH9， MYCT1， HKDC1， CFAP43， RAB11FIP1， PREX2， SCUBE1， CCDC68， ADAM33， SLC19A3， KIAA1683， SLC44A4， ITIH5， UPK3B， APOL3， TUBB1， KCNH6， COLEC12， SYNC， DCSTAMP， APOLD1， COL21A1， TMEM163， CAB39L， DOCK8， SPRY4， ST6GALNAC5， DRC3， TTC25， SOX7， FAM167A， DYNLRB2， TEKT1， MS4A8， FERMT3， SYT15， ESYT3， ROPN1L， USHBP1， SNX25， TTC29， USP44， PRAM1， LRRIQ1， PPP1R1B， ZMYND15， DRC7， ATP13A4， C2orf40， ADGRA1， ZMAT1， MEGF11， FBN3， JPH4， HOPX， PARD6B， SLITRK2， PLA2G12B， ADGRE3， C9orf24， MS4A14， COX4I2， ADTRP， PTPN5， RSPO3， MFSD2A， ATOH8， CGNL1， MICALCL， TOX2， DISP1， RERG， LGALS12， KNDC1， CCDC65， DNAJC5B， RSPH1， WNT3A， KCNK17， MVB12B， MAATS1， FAM181A， CEACAM21， EFCAB12， LRRC46， STARD13， N4BP2L1， IL33， ESAM， IGFN1， GGTLC2， PKDCC， ANKRD44， SLFN11， NEXN， NLRP12， MYADM， ZNF804A， CABLES1， MYLK3， CHRDL1， GGTLC1， TMEM88， DUSP27， RCSD1， TMEM132C， SCGB3A1， PRR29， DRC1， B3GNT7， PKHD1L1， C1orf158， MYOCD， ARHGAP18， CADPS2， CLEC6A， PPP1R14A， UCN3， SIGLEC11， NLRP3， CARD16， BTBD9， KLHL32， ELFN2， CCDC85A， C1QTNF2， C1QTNF7， SLC26A9， KCTD12， GPR146， GBP4， FAM46B， NOSTRIN， RASGRP4， PHACTR3， CYYR1， CMTM5， ACSM1， C8orf34， TM4SF18， OLIG1， HSPA12B， WDR17， CATSPER1， MIA2， SCGB3A2， SH2D1B， HRASLS5， IP6K3， TMC2， PARD3B， AGAP11， FAT3， JAML， LRRK2， TMEM132D， ERP27， FGD4， SLAIN1， TDRD9， AK7， SLC24A4， SLC51B， AGBL1， DNAAF1， KIF19， WFIKKN2， SPNS2， LOXHD1， OSCAR， GIPC3， MISP， HSPB6， TDRD10， SHE， WDR63， C1orf194， ERICH3， KLHDC7A， UBXN10， C1orf87， GOLT1A， KLF17， TMEM125， PIFO， C1orf162， WFDC12， C20orf85， SIRPD， SGSM1， TRABD2A， C2orf73， CCDC173， PLEKHH2， ALS2CR12， TMEM178A， TRIM71， COL6A6， SNTN， SPATA18， SPATA4， CAPSL， ZNF474， C5orf49， AFAP1L1， PACRG， DPCR1， TMEM139， C7orf57， C9orf135， PIH1D3， PTCHD1， GAB3， DOCK11， TCEAL2， GATA5， CBLN4， CCM2L， MACROD2， ITLN2， DYDC1， MUC15， GLT1D1， TSPAN19， FAM216B， PPP1R36， GCOM1， VWA3A， CMTM2， TMED6， C16orf89， CD300LF， CFAP52， UNC45B， CD300LG， CCBE1， ANKRD29， TMEM190， MFSD4A， PM20D1， GCSAML， DCDC2B， CFAP57， CCDC17， LRRC71， FAM71A， C22orf15， NFAM1， TEKT4， ZNF385B， GPBAR1， EFHB， WDR49， GLIPR2， ZCWPW2， NEK10， CCDC13， FGD5， CNTN4， SHISA3， KLB， RASGEF1B， BTNL9， FAM81B， CFAP206， IQUB， TMEM213， GIMAP8， AGR3， PEBP4， SBSPON， LCN6， TTC16， FREM1， AKAP14， AWAT2， ZDHHC15， CFAP58， CLEC12A， GRASP， SLC5A8， CCDC60， STOML3， CLEC14A， CFAP161， SLC16A11， LGI4， LRRN4， TTLL9， DAW1， LONRF2， FAM179A， CLEC4F， PRICKLE2， SGMS2， NIM1K， ZNF366， PRSS35， C6orf118， GIMAP7， BMPER， C10orf128， OIT3， HTR3C， GIMAP1， SYNPO2， PTCRA， CYS1， SDR16C5， DNAH10， VSTM4， ADCY4， GRAMD2， MCEMP1， C1orf168， SLC5A9， TCTEX1D1， SLFNL1， CFAP221， VWA3B， GKN2， LIPH， CEP112， UNC13D， DNAH12， RANBP3L， GAPT， TCTE1， KHDRBS2， LGI3， ANO5， PLAC9， C10orf107， ENKUR， ARMC3， RTKN2， MPEG1， MS4A15， PPP1R32， DOK6， OTUD1， FAM13C， SPATA13， FAM162B， ADGRF5， SPATS1， RSPH9， KIF6， FGD2， PI16， RNF182， PHACTR1， MTURN， KIAA1324L， CDHR3， NKAPL， TMEM130， GAS2L2， MSRB3， ANGPTL5， SFTA3， TTLL10， PHYHD1， MORN5， CFAP65， C4orf22， PLA2G4F， NPNT， C19orf38， BCL6B， COL6A5， ST6GALNAC3， MAMDC2， C10orf67， KANK3， FRMD3， CATSPERD， ARHGAP30， CFAP126， NEGR1， MAGI3， SEC14L3， OLFML1， RASSF3， ADGRD1， PRTG， SAXO2， EXOC3L1， C16orf54， NHLRC4， TTLL6， PTRF， CXCL17， TMEM150B， RIIAD1， RSPO1， SIRPB2， SEC14L4， FAM150B， CCDC141， IGSF10， CYP4V2， DOK7， GPRIN3， RNF180， PPIL6， SCARA5， CFAP157， RAB37， GPIHBP1， CLEC4D， FFAR4， ST8SIA6， FAM92B， LINGO4， BRINP3， C1orf228， TREML1， RSPO2， OVCH2， OVCH1， NEK5， GLDN， CD300E， SMTNL2， KRT27， ZNF677， PALM3， OR2W3， MYBPHL， FCRL6， KCNT2， TCTEX1D4， RSPO4， KIAA1211L， NCKAP5， HSD17B13， PLCXD3， RSPH4A， ECT2L， CFAP100， SLCO4C1， LILRA5， TBC1D10C， PTPRQ， CCDC178， C19orf35， PEAR1， CATIP， LHFPL3， PNPLA7， KLHL30， SOGA3， SH2D4B， SLC16A12， INSC， FIBIN， C11orf96， CFAP73， TMEM233， C15orf52， C2CD4B， SCIMP， TMEM220， LDLRAD1， C1orf189， CAPN8， C2orf71， CDHR4， MUSTN1， VGLL3， TMEM212， SFTA2， IYD， FAM180A， CFAP77， MAP3K15， LRRC10B， GLOD5， MUC21， C11orf88， SERTM1， FAM174B， NCMAP， C1orf141， C20orf202， RGS7BP， HACD4， C9orf152， SNX30， WDR38， APOBEC4， ERVFRD-1， TNFSF12-TNFSF13， LCN10， IQSEC3， SMCO3， PLIN5， FAM183A， C1orf186， MAP1LC3C， AARD， TARP， PALM2-AKAP2， GIMAP6， LRRC18， ECSCR， TMEM232， KIAA0754， LIPN， SYNDIG1L， HRCT1， FAM228A， SFTPA1， TMEM236， RSPH10B2， DEFA1B， DMRTC1B， SFTPA2， PSAPL1， SIGLEC14， FAM47E， SMIM6， ANKRD66 |
| **Up** | ASIC1， ACTL6A， ADH7， JAG1， NR0B1， AK4， ALDH3B2， ALOX12B， BIRC5， ASCL2， ASNS， ATP2A1， KIF1A， ADGRB2， NKX3-2， HCN2， BIK， BLM， BNC1， BMP7， FOXL2， BRCA1， BUB1， BUB1B， MPPED2， MPPED1， CA9， CA12， CACNA1B， CALB1， CALML3， CBR3， CCNA2， CCNB1， CCNE1， CCNF， ENTPD2， CDK1， CDC6， CDC20， CDC25A， CDC25C， CDH3， CDH8， CDKN2A， CDKN3， CDX1， CEL， CENPA， CENPE， CENPF， RCC1， CHEK1， CHGB， CHRM3， CHRNA5， CHRNB2， CHRNB4， CKMT1B， CKS1B， CKS2， CLCN2， COL1A1， COL2A1， COL3A1， COL4A6， COL5A2， COL7A1， COL9A3， COL10A1， COL11A1， COL17A1， CRABP1， CRABP2， SLC25A10， CST1， CST4， CSTA， NKX2-5， CTSV， CYP24A1， CYP26A1， CYP27B1， AKR1C1， AKR1C2， DDX11， TIMM8A， COCH， DIO2， DLX1， DLX2， DLX5， DLX6， DNA2， DYNC1I1， DNMT3B， DRD4， DRP2， DSC1， DSC2， DSC3， DSG1， DSG2， DSG3， DSP， EPYC， DTYMK， DUSP9， E2F1， E2F2， ECT2， EEF1A2， EFNA3， EFNA4， CELSR3， CELSR2， EIF4EBP1， EMX1， EN1， EN2， EPHB2， EPHB3， ETV4， EYA2， EZH2， F2RL2， F12， FABP7， FANCA， FANCD2， FANCE， FANCB， FANCG， FAT2， FBN2， FEN1， FGF11， FKBP4， FOXD1， FOXE1， FOXM1， CENPI， FUT2， GABRA3， GABRR1， GAD1， B4GALNT1， GAP43， GAPDH， GCSH， GJA3， GJB2， GJB3， GJB5， GCLC， GNB3， GNG4， GNGT1， SFN， GPC1， NPBWR1， GPR19， GPX2， GRIN2D， GRM4， GUCA1A， HIST1H1D， HIST1H1E， HIST1H2AE， H2AFX， HIST1H2BD， HELLS， HMGB3， HMGA1， HMMR， HNF4G， HOXA1， HOXA10， HOXA11， HPCA， HOXB7， HOXB8， HOXB9， HOXC4， HOXC5， HOXC6， HOXC8， HOXC9， HOXC10， HOXC11， HOXC13， HOXD3， HOXD9， HOXD10， HOXD11， HOXD13， HSD17B1， HSD17B3， HTR2C， HTR3A， HTR7， IBSP， IGFBP2， IGFBP3， IGSF1， IL11， IL12RB2， INCENP， INHA， IRF6， ISL1， ITGB4， ITPKA， IVL， KCNA7， KCND2， KCNG1， KCNH1， KCNJ10， KIF3C， KIF5A， KIF11， KIFC1， KPNA2， KRT5， KRT6A， KRT6B， KRT13， KRT14， KRT15， KRT16， KRT17， KRT31， KRT32， KRT34， KRT81， LAD1， LGALS7， LMNB1， LMO1， LMX1B， CYP4F3， MAD2L1， MAGEA1， MAGEA2， MAGEA3， MAGEA4， MAGEA6， MARK1， MCM2， MCM4， MCM6， MCM7， ADAM11， MDFI， MDK， MFAP2， MELTF， MIF， MKI67， MMP1， MMP3， MMP9， MMP10， MMP11， MMP12， MMP13， MSI1， NUDT1， MYBL2， MYCL， MYCN， NEB， NEFL， NEK2， TONSL， NKX6-1， NME1， NRCAM， NTS， ORC1， OTX1， OVOL1， PAFAH1B3， PAX7， PAX9， PCNA， PCSK1， PDK1， PFN2， PGF， PI3， SERPINB5， SERPINE2， SERPINB13， PITX1， PITX2， PKP1， PKP2， PLAU， PLK1， PLOD2， PLXNB3， PMAIP1， POLE2， POLR2H， POU3F1， POU4F1， PPAT， PPEF1， PPP2R2C， PRB3， PRIM2， PROC， PRSS3， KLK6， PSPH， PTGFRN， PTHLH， PTH2R， PTPRH， PTPRN， PTPRZ1， NECTIN1， PYCR1， RAB3B， RAC3， RAD51， RANBP1， RFC4， RFC5， RPL36A， RRM2， S100A2， S100A7， SALL1， SERPINB3， SERPINB4， SCN8A， SCG5， SHMT2， SHOX2， STIL， SIM2， SIX1， SKP2， SLC4A3， SLC1A4， SLC2A1， SLC2A5， SLC6A2， SLC6A8， SLC6A11， SLC9A2， SLC9A3， SLC15A1， SLC16A1， SNAI2， SMO， FSCN1， SORD， SOX2， SOX11， SOX15， SPOCK1， SPP1， SPRR1A， SPRR1B， SPRR2A， SPRR2D， SPRR2E， SPRR2G， SPRR3， SPTBN2， SRD5A1， STAR， ELOVL4， AURKA， STX1A， SYT1， SYT5， TBX1， TCF19， TCN1， TCTE3， TERT， TFAP2A， TFAP2C， TFAP4， TFR2， TG， THBS2， TK1， TLL2， TNNT1， TOP2A， TP73， TPBG， TPD52L1， TPI1， TTK， TWIST1， TYMS， UCHL1， UPK1B， UGT8， UCK2， UNG， VGF， VSNL1， WNT10B， XDH， XG， XRCC2， XRCC3， ZIC1， ZIC2， MKRN3， ZP3， LRP8， ADAM12， HMGA2， AP3B2， ST8SIA2， SLC7A5， DPF1， CHAF1B， CDC7， CDC45， FZD9， HIST1H2AM， HIST1H2BG， HIST1H2BH， HIST1H2BO， HIST1H3D， HIST1H3E， HIST1H3G， HIST1H3H， HIST1H4J， HIST1H4E， HIST2H4A， BBOX1， RASAL1， RAD54L， FOXN1， PPFIA4， BARX2， PDLIM4， LY6D， TNFSF11， RGS20， RDH16， PLPP2， TP63， SERPINB7， TNFRSF25， ADAM23， TNFRSF18， IL1RL2， GGH， WISP3， WISP1， CDK5R1， SPHK1， SLC5A6， TIMELESS， FOXH1， WASF1， CDK5R2， HIST1H2AG， HIST1H2BJ， TRPA1， HAP1， RHBDL1， ARTN， PRC1， CLDN1， PKMYT1， TBX18， INA， KRT75， CCNB2， CCNE2， SYNGR3， EXO1， AURKB， PTTG1， TRIP13， TGM5， LHX2， RECQL4， TMPRSS11D， ECEL1， AIM2， ONECUT2， KIF23， STXBP5L， PTGES， CXCL14， APOBEC3B， KIF20B， GDA， NUP155， TESMIN， CLCA2， PLCH2， GREB1， RIMS2， ESPL1， ECE2， KNTC1， KIAA0101， DLGAP5， ARHGAP11A， MELK， GINS1， SV2A， NCAPD2， KIF14， XYLB， HS3ST3A1， BCL2L10， TROAP， CHAF1A， ABCC5， PPIF， KIF20A， ALG3， DDX39A， TRAIP， B3GALT5， B3GNT3， CCL26， NPM3， TUBB3， SCML2， NDC80， FST， HOXB13， C1orf61， SEMA4B， CIB2， RNASEH2A， OLFM4， CXCL13， DPYSL4， PAICS， P3H4， SPAG5， RAD51AP1， IGF2BP1， IGF2BP3， CGREF1， DLL3， POLQ， PLK4， SIX2， PLAC1， MTHFD2， RPP40， GJB6， FTCD， NMU， C1QL1， RUNDC3A， DBF4， MLLT11， KIF2C， UBE2C， HSF2BP， ZWINT， DMC1， SOX21， WDHD1， PKP3， ANXA10， CHEK2， KLK8， FZD10， NXPH4， POU6F2， OIP5， ITGA11， CLCA4， HSPA4L， CCT5， TPX2， AKR7A3， MAST1， HECW1， DDN， ATP10B， TTLL12， SULF1， BOP1， KIAA1024， NCAPH， GRIP1， PRAME， SLC16A8， ORC6， CBLC， TRIM29， SLC7A11， KIF4A， FJX1， KIAA1549L， RAD54B， NGEF， VAX2， SULT4A1， RAB26， POC1A， TMEM158， KRT23， TSKU， TENM4， CNTNAP2， ERC2， ABCA12， PHGDH， CABYR， FAM162A， PPP1R14B， PLEK2， IL36RN， RGS17， SRPK3， MYEOV， GREM1， CKAP2， STEAP1， FOXD3， LYPD3， MTBP， FAM155B， SMC1B， CPNE7， ADAMDEC1， UBE2S， SLCO1B3， ATAD2， UBE2T， C6orf15， PRSS50， RACGAP1， UHRF1， GRHL1， PSMC3IP， SAC3D1， HILPDA， PADI1， PNMA3， PSAT1， DONSON， SLC39A2， ERO1A， PODXL2， IRX4， FOXP3， SOST， PI15， GMNN， GAL， YBX2， RAPGEFL1， CPA4， NUSAP1， DUSP13， KCNK9， REEP2， WNT16， PRRX2， RHCG， VCX3A， GTSE1， DTL， IL23A， GINS2， MPP6， PADI3， SIX4， COQ3， CALML5， BCL11A， S1PR5， IL20RB， GPR87， MIS18A， ANLN， FAM64A， SMOX， PUS7， NECAB2， UGT1A7， UGT1A6， GNB1L， UGT1A9， HES2， UGT1A1， CNGB3， LY6K， DIRAS2， ERCC6L， GDPD2， NCAPG2， ELOVL2， CYP2W1， RHBDL2， SOHLH2， TMEM132A， TTC22， PARPBP， CDCA4， EPN3， ZWILCH， AIM1L， SUSD4， KIF26B， SLC6A15， CDCA8， CEP55， NUDT11， PLEKHG6， FANCI， ETNK2， NEIL3， TMEM40， BRIX1， HJURP， STYK1， LGR4， MCM10， HES6， FERMT1， DEPDC1， NDC1， CEP72， ASF1B， ASUN， C1orf112， OGDHL， WDR12， PRR11， DEPDC1B， HR， ADCY10， CENPN， PBK， GABRQ， RCC2， SEPT3， BARX1， GSDMC， CPXM1， IL36G， KCNQ5， EIF5A2， TMPRSS4， C10orf2， PANX2， NRIP3， EXOSC5， ARNTL2， RGMA， KIF15， AKR1B10， CASC5， PGLYRP4， ZNF695， TMEM63C， SALL4， VANGL2， TTYH1， SPC25， TENM2， CASKIN1， PCDH19， IGSF9， SYT13， KIAA1524， GRHL3， CYP4F11， DMRT3， IL22RA1， ALOXE3， CHTF18， CHP2， DMRTA2， CLSPN， TP53AIP1， FIGNL1， PERP， SLC28A3， CENPK， NCAPG， POPDC3， LHX5， STRA6， CDH24， GINS3， ISL2， CENPH， MRPL36， SLC26A10， ZFP69B， WNK2， DLK2， RASL11B， C17orf53， LRFN4， AUNIP， CENPM， TRPM8， DSCC1， CARD14， CENPO， FSD1， B3GNT4， KREMEN2， ULBP3， NKAIN1， GALNT14， VTCN1， CENPU， SUV39H2， E2F8， ZNF750， SUGCT， SHCBP1， AGMAT， BORA， PODNL1， ATAD5， ADM2， MAP6D1， CNTD2， SYNDIG1， DNAJC22， POF1B， ADAMTS20， BAIAP2L2， PIF1， ZNF703， C16orf59， MYO19， TRIM45， TET1， ULBP2， PABPC1L， LY6G6C， PRR7， TMEM177， MAGED4B， NECTIN4， FAM83D， CDT1， NETO2， NETO1， KIF18A， CDCA3， NUF2， ESPN， INHBE， CDCA7， GSG2， SLC4A11， BRIP1， MND1， KIRREL2， PLEKHN1， HORMAD1， TMEM117， TMEM79， CAPNS2， GINS4， CMSS1， THOC3， BRSK1， MEGF10， MCM8， CNFN， ZC3H8， C15orf41， PARD6G， FNDC1， TTBK1， KISS1R， RNASE7， SLC9A7， GPT2， PSRC1， CBX2， TUBA1C， C19orf48， PERM1， LMNB2， HPDL， LINGO1， ARHGEF39， TNS4， FAM83A， KRTAP4-1， ZIC5， STON2， EPT1， LBX2， ARHGAP11B， SLAMF9， SAPCD2， HS6ST2， UCN2， KLHL13， TICRR， KNSTRN， MLIP， CCDC34， RCCD1， FAAP24， RASL10B， DEPDC7， SYT12， CENPL， MARVELD3， ZC3HAV1L， DAPL1， CDHR1， MEX3A， MGME1， HIST3H2A， LEMD1， TBC1D31， CGB7， CAMK2N2， EGLN3， MRAP2， MTFR2， CDCA5， AHNAK2， FAM83F， TRIM9， PGLYRP3， GPRIN1， C1QTNF6， FBXO32， TNFRSF13C， ALPK2， C12orf56， CTHRC1， RMI2， LYPD1， IL22RA2， RPL39L， MED12L， PRAP1， ZNF488， ANKRD22， GYLTL1B， C12orf54， KRT74， BTBD11， FRMD6， PAQR4， USH1G， ANKRD13B， FAM69C， COX6B2， FBXO27， ANKLE1， RPTN， HIST2H3C， DMBX1， GJB4， DNAH14， EDARADD， TSACC， IQGAP3， ARHGEF19， FAM83C， ACVR1C， TMEM182， CCDC58， LRRC15， ARL9， IL31RA， UBE2QL1， TMEM171， SRSF12， PNCK， MYO3B， CHODL， CTCFL， BRI3BP， ADAMTS14， SRXN1， RBBP8NL， OR51E1， SESN3， FAM101A， WDR66， E2F7， A2ML1， ISM2， C2CD4A， SLC47A2， KIF18B， EME1， TRIM16L， CBLN2， KLC3， SPC24， IGFL2， CILP2， C1orf74， CIART， SLC35F3， WFDC5， YDJC， CKAP2L， PROM2， SGO2， FAM84A， SGO1， XXYLT1， IGSF11， PLEKHG4B， RAET1L， NKAIN2， ABCA13， CLEC2L， VPS37D， CDCA2， ESCO2， C9orf84， SLC5A12， STRC， TRPV3， GBP6， TDRD5， SASS6， APCDD1L， CCDC138， DQX1， SPTSSB， COL22A1， SNX31， C10orf91， NUDT10， ADAMTS18， RHOV， ZNF367， PLPP4， KRT78， CREG2， ANKRD23， ALG1L， C3orf67， FBXO45， RDM1， TMEM184A， ADCK5， ADAM32， CERS3， SLC44A5， GLYATL2， DDIAS， SPERT， SKA1， FAM181B， SKA3， BEND6， ADGRF4， VWDE， SP8， GPC2， SRRM3， FAM83B， SLC29A4， ATP6V1C2， CNIH2， CBARP， SYT14， STEAP1B， MAP7D2， WDR72， NPB， ASPM， AQP11， RCOR2， NPW， FADS6， FAM171A2， SLC13A5， LAMA1， PRR19， TMEM145， NKPD1， KLK9， WDR62， SMIM24， CCDC150， KIF4B， ATG9B， FBXO43， FAM133A， LIN9， TRIM59， KRT6C， PPM1J， S100A7A， B4GALNT4， C1QL4， RNASE10， PRSS53， TMEM52， CYP27C1， TMPRSS11A， NAT8L， DCAF12L2， LHFPL1， HEPHL1， SMCO2， NCCRP1， KIF24， ITIH6， TUBB2B， FAM131C， GEN1， C2orf48， NIPAL4， BMP8A， FAM111B， KRT77， SBSN， IGFL1， C5orf34， KCP， CENPW， C10orf99， KRTDAP， FAM132A， MFSD2B， SLC9A4， TMPRSS11F， C5orf46， FEZF1， CSAG3， ACP7， FRRS1， SH2D5， EML6， AMTN， CENPP， IGLON5， C16orf74， C10orf55， SLC22A20， ZYG11A， CAPN14， AKR1B15， NRARP， CHAC2， TYMSOS， CKMT1A， MIR205HG， TMEM200C， LGALS7B， PHLDB3， FAM72B， MAGED4， MAGEA9B， CCDC169， FAM72D， TP53TG3B， RAD51AP2， FAM72A， LRRC69， UPK3BL， C17orf99， C17orf96 |
