# Supplementary material for: Integrated analysis of competing endogenous RNA network revealing lncRNAs as potential prognostic biomarkers in human lung squamous cell carcinoma
Source: Oncotarget. 2017 Jul 27;8(39):65997–6018. doi: 10.18632/oncotarget.19627 (PMC5630388; doi:10.18632/oncotarget.19627)
Supplement: Supplementary file 3 [file oncotarget-08-65997-s003.docx]

**Supplementary Table 4: Dysregulated KEGG pathway and GO term in LUSC**

| **Category** | **ID** | **Term** | **No. of genes** | **-lgP** | **FDR** |
| --- | --- | --- | --- | --- | --- |
| **KEGG pathway** | 05202  04974  05200  05205  04550  04727  04060  04723  04726  04151  04530  04010  04921  04012  05032  05206  04713  04725  04724  04728  04380  04260  04925  04020  04916  05146  04960  04071  04930  04310  04978  04742  03460  04261  04390  04630  04022  04610  04918  04976  04062  05412  04024  04510  05410  04350  04015  04911  05414  04064  04912  04014  04915  04144  05166  04931  04080  04270  05033  04973  01100  00600  04961  04340  05217  05214  04720  05031  04917  05220  04971  05222  04512  04640  04970  05323  04666  04972  04750  04066  04919 | Transcriptional misregulation in cancer  Protein digestion and absorption  Pathways in cancer  Proteoglycans in cancer  Signaling pathways regulating pluripotency of stem cells  GABAergic synapse  Cytokine-cytokine receptor interaction  Retrograde endocannabinoid signaling  Serotonergic synapse  PI3K-Akt signaling pathway  Tight junction  MAPK signaling pathway  Oxytocin signaling pathway  ErbB signaling pathway  Morphine addiction  MicroRNAs in cancer  Circadian entrainment  Cholinergic synapse  Glutamatergic synapse  Dopaminergic synapse  Osteoclast differentiation  Cardiac muscle contraction  Aldosterone synthesis and secretion  Calcium signaling pathway  Melanogenesis  Amoebiasis  Aldosterone-regulated sodium reabsorption  Sphingolipid signaling pathway  Type II diabetes mellitus  Wnt signaling pathway  Mineral absorption  Taste transduction  Fanconi anemia pathway  Adrenergic signaling in cardiomyocytes  Hippo signaling pathway  Jak-STAT signaling pathway  cGMP-PKG signaling pathway  Complement and coagulation cascades  Thyroid hormone synthesis  Bile secretion  Chemokine signaling pathway  Arrhythmogenic right ventricular cardiomyopathy (ARVC)  cAMP signaling pathway  Focal adhesion  Hypertrophic cardiomyopathy (HCM)  TGF-beta signaling pathway  Rap1 signaling pathway  Insulin secretion  Dilated cardiomyopathy  NF-kappa B signaling pathway  GnRH signaling pathway  Ras signaling pathway  Estrogen signaling pathway  Endocytosis  HTLV-I infection  Insulin resistance  Neuroactive ligand-receptor interaction  Vascular smooth muscle contraction  Nicotine addiction  Carbohydrate digestion and absorption  Metabolic pathways  Sphingolipid metabolism  Endocrine and other factor-regulated calcium reabsorption  Hedgehog signaling pathway  Basal cell carcinoma  Glioma  Long-term potentiation  Amphetamine addiction  Prolactin signaling pathway  Chronic myeloid leukemia  Gastric acid secretion  Small cell lung cancer  ECM-receptor interaction  Hematopoietic cell lineage  Salivary secretion  Rheumatoid arthritis  Fc gamma R-mediated phagocytosis  Pancreatic secretion  Inflammatory mediator regulation of TRP channels  HIF-1 signaling pathway  Thyroid hormone signaling pathway | 9  7  10  8  7  6  8  6  6  8  6  7  6  5  5  7  5  5  5  5  5  4  4  5  4  4  3  4  3  4  3  3  3  4  4  4  4  3  3  3  4  3  4  4  3  3  4  3  3  3  3  4  3  4  4  3  4  3  2  2  7  2  2  2  2  2  2  2  2  2  2  2  2  2  2  2  2  2  2  2  2 | 10.674  9.609  8.926  8.629  8.210  7.868  7.720  7.506  7.235  6.832  6.693  6.457  6.344  6.160  6.062  6.009  5.969  5.632  5.574  5.308  5.275  4.700  4.635  4.612  4.271  4.139  4.011  3.959  3.738  3.686  3.659  3.634  3.609  3.604  3.537  3.494  3.401  3.266  3.230  3.230  3.212  3.176  3.109  3.044  3.029  3.013  3.012  2.998  2.939  2.911  2.911  2.892  2.804  2.682  2.682  2.681  2.567  2.560  2.222  2.120  2.118  2.083  2.083  2.012  1.948  1.806  1.793  1.780  1.719  1.707  1.696  1.569  1.559  1.559  1.540  1.540  1.513  1.477  1.460  1.418  1.306 | 0.000  0.000  0.000  0.000  0.000  0.000  0.000  0.000  0.000  0.000  0.000  0.000  0.000  0.000  0.000  0.000  0.000  0.000  0.000  0.000  0.000  0.000  0.000  0.000  0.000  0.000  0.001  0.001  0.001  0.001  0.001  0.001  0.001  0.001  0.001  0.001  0.002  0.002  0.002  0.002  0.002  0.002  0.003  0.003  0.003  0.003  0.003  0.003  0.004  0.004  0.004  0.004  0.005  0.006  0.006  0.006  0.007  0.007  0.016  0.019  0.019  0.020  0.020  0.024  0.027  0.037  0.037  0.038  0.043  0.043  0.044  0.058  0.058  0.058  0.059  0.059  0.062  0.066  0.068  0.074  0.095 |
| **GO** | GO:0070120  GO:0007268  GO:0043524  GO:0045944  GO:0008284  GO:0043066  GO:0030574  GO:0045087  GO:0022617  GO:0007420  GO:0048661  GO:0007411  GO:0043627  GO:0045669  GO:0008217  GO:0048011  GO:0035556  GO:0030316  GO:0002027  GO:0038165  GO:0030182  GO:0045893  GO:0042517  GO:0043507  GO:0006468  GO:0048861  GO:0055085  GO:0006112  GO:0030198  GO:0008016  GO:0007165  GO:0042127  GO:0030335  GO:0044281  GO:0007605  GO:0031100  GO:0034613  GO:0003139  GO:0086004  GO:0045892  GO:0045165  GO:0007155  GO:0006898  GO:0007507  GO:0071300  GO:0007399  GO:0070102  GO:0034097  GO:0000082  GO:0014902  GO:0046620  GO:0090303  GO:0000122  GO:0043410  GO:0060384  GO:0008283  GO:0007389  GO:0051216  GO:0086005  GO:0006874  GO:0006629  GO:0007173  GO:0001503  GO:0051091  GO:0006978  GO:0042953  GO:0002548  GO:0010881  GO:0045672  GO:0060749  GO:0090090  GO:0010634  GO:0019229  GO:0019221  GO:0030224  GO:0031290  GO:0007214  GO:0045736  GO:0007049  GO:0010575  GO:0035249  GO:0060045  GO:0006357  GO:0009314  GO:0051899  GO:0051092  GO:0001958  GO:0007417  GO:0006461  GO:0043552  GO:0051924  GO:0006897  GO:0009615  GO:0009790  GO:0034446  GO:0048701  GO:0034220  GO:0055010  GO:0007528  GO:0030318  GO:0051301  GO:0010628  GO:0050853  GO:0007628  GO:0071560  GO:0032024  GO:0032755  GO:0048538  GO:0007186  GO:0007611  GO:0043123  GO:0050830  GO:0001974  GO:0006956  GO:0008542  GO:0021766  GO:0003151  GO:0001755  GO:0051260  GO:0002651  GO:0002663  GO:0010464  GO:0010536  GO:0010743  GO:0014813  GO:0032509  GO:0032946  GO:0035585  GO:0036371  GO:0045713  GO:0048537  GO:0051545  GO:0060538  GO:0060611  GO:0060668  GO:0060694  GO:0061026  GO:0061301  GO:0070104  GO:0070106  GO:0070512  GO:0086066  GO:0090038  GO:1901021  GO:1901258  GO:2000097  GO:2000108  GO:2000617  GO:2000620  GO:2001031  GO:0001568  GO:0030199  GO:0048469  GO:0008543  GO:0016055  GO:0048704  GO:0006810  GO:0033554  GO:0006811  GO:0007588  GO:0008285  GO:0007519  GO:0031398  GO:0045665  GO:0006633  GO:0006367  GO:0006915  GO:0007269  GO:0000902  GO:0000079  GO:0018108  GO:0002066  GO:0002384  GO:0003006  GO:0003138  GO:0003185  GO:0006677  GO:0006682  GO:0007196  GO:0010882  GO:0010899  GO:0021551  GO:0032680  GO:0032730  GO:0033292  GO:0034112  GO:0035408  GO:0035669  GO:0035984  GO:0036309  GO:0038180  GO:0042488  GO:0042701  GO:0043482  GO:0046168  GO:0046600  GO:0048227  GO:0048936  GO:0050925  GO:0051466  GO:0051900  GO:0060025  GO:0060297  GO:0060434  GO:0060443  GO:0061041  GO:0071657  GO:0071812  GO:0071847  GO:0071848  GO:0072539  GO:0086015  GO:0086046    GO:0090074  GO:1901018  GO:1901019  GO:2001016  GO:2001295  GO:0001523  GO:0007603  GO:0030900  GO:0042472  GO:0001570  GO:0060021  GO:0007169  GO:0007584  GO:0045666  GO:0002821  GO:0007207    GO:0007521  GO:0008582  GO:0009048  GO:0009750  GO:0010829  GO:0010871  GO:0014051  GO:0021524  GO:0021891  GO:0031987  GO:0032966  GO:0035426  GO:0042415  GO:0043009  GO:0043415  GO:0044211  GO:0045652  GO:0045916  GO:0045988  GO:0046058  GO:0048625  GO:0048880  GO:0050861  GO:0050891  GO:0051572  GO:0051574  GO:0051586  GO:0051932  GO:0055098  GO:0060005  GO:0060024  GO:0060083  GO:0060379  GO:0060440  GO:0061304  GO:0061333  GO:0061364  GO:0070257  GO:0070296  GO:0070508  GO:0070779  GO:0071322  GO:0071453  GO:0071864  GO:0072095  GO:0072102  GO:0072160  GO:0086070  GO:2000366  GO:2001013  GO:0051897  GO:0007596  GO:0060070  GO:0042593  GO:0006814  GO:0050796  GO:0002175  GO:0002666  GO:0003266  GO:0006084  GO:0008593  GO:0014910  GO:0021559  GO:0030947  GO:0035357  GO:0038031  GO:0044030  GO:0046323  GO:0051138  GO:0051409  GO:0051451  GO:0055005  GO:0060336  GO:0060415  GO:0060596  GO:0060913  GO:0061299  GO:0061337  GO:0070972  GO:0072107  GO:2000010    GO:2000111  GO:2000987  GO:2001259  GO:0071456  GO:0038096  GO:0045766  GO:0016337  GO:0006813  GO:0007517  GO:0002446  GO:0002634  GO:0002768  GO:0003283  GO:0003360  GO:0006534  GO:0006686  GO:0006734  GO:0009410  GO:0010891  GO:0014909  GO:0015670  GO:0016199  GO:0021889  GO:0022402  GO:0033081  GO:0033993  GO:0035278  GO:0040011  GO:0042538  GO:0043269  GO:0043619  GO:0044206  GO:0045657  GO:0046321  GO:0048311  GO:0050764  GO:0050996  GO:0051005  GO:0051549  GO:0051938  GO:0051964  GO:0060763  GO:0070295  GO:0071374  GO:0071498  GO:0071681  GO:0086064  GO:0006281  GO:0009653  GO:0007243  GO:0007568  GO:0001504  GO:0001867  GO:0002281  GO:0003211  GO:0007098  GO:0007182  GO:0007223  GO:0010694  GO:0010887  GO:0015909  GO:0021520  GO:0021895  GO:0030643  GO:0030913  GO:0032717  GO:0032725  GO:0034259  GO:0045822  GO:0048643  GO:0048752  GO:0050810  GO:0051150  GO:0051497  GO:0051573  GO:0060044  GO:0060463  GO:0090073  GO:1900153  GO:0006486  GO:0002674  GO:0006301  GO:0010740  GO:0010763  GO:0014033  GO:0019226  GO:0019227  GO:0030857  GO:0035641  GO:0042117  GO:0048762  GO:0051279  GO:0051481  GO:0051974  GO:0055012  GO:0060119  GO:0060213  GO:0060373  GO:0060536  GO:0070723  GO:0071361  GO:0085020  GO:0086014  GO:2000727  GO:0008360  GO:0000002  GO:0001763  GO:0006359  GO:0016322  GO:0030252  GO:0030278  GO:0030299  GO:0031325  GO:0032731  GO:0034383  GO:0035313  GO:0045446  GO:0045651  GO:0045670  GO:0048541  GO:0071549  GO:0072075  GO:0030308 | ciliary neurotrophic factor-mediated signaling pathway  synaptic transmission  negative regulation of neuron apoptotic process  positive regulation of transcription from RNA polymerase II promoter  positive regulation of cell proliferation  negative regulation of apoptotic process  collagen catabolic process  innate immune response  extracellular matrix disassembly  brain development  positive regulation of smooth muscle cell proliferation  axon guidance  response to estrogen stimulus  positive regulation of osteoblast differentiation  regulation of blood pressure  neurotrophin TRK receptor signaling pathway  intracellular signal transduction  osteoclast differentiation  regulation of heart rate  oncostatin-M-mediated signaling pathway  neuron differentiation  positive regulation of transcription, DNA-dependent  positive regulation of tyrosine phosphorylation of Stat3 protein  positive regulation of JUN kinase activity  protein phosphorylation  leukemia inhibitory factor signaling pathway  transmembrane transport  energy reserve metabolic process  extracellular matrix organization  regulation of heart contraction  signal transduction  regulation of cell proliferation  positive regulation of cell migration  small molecule metabolic process  sensory perception of sound  organ regeneration  cellular protein localization  secondary heart field specification  regulation of cardiac muscle cell contraction  negative regulation of transcription, DNA-dependent  cell fate commitment  cell adhesion  receptor-mediated endocytosis  heart development  cellular response to retinoic acid  nervous system development  interleukin-6-mediated signaling pathway  response to cytokine stimulus  G1/S transition of mitotic cell cycle  myotube differentiation  regulation of organ growth  positive regulation of wound healing  negative regulation of transcription from RNA polymerase II promoter  positive regulation of MAPK cascade  innervation  cell proliferation  pattern specification process  cartilage development  regulation of ventricular cardiac muscle cell action potential  cellular calcium ion homeostasis  lipid metabolic process  epidermal growth factor receptor signaling pathway  ossification  positive regulation of sequence-specific DNA binding transcription factor activity  DNA damage response, signal transduction by p53 class mediator resulting in transcription of p21 class mediator  lipoprotein transport  monocyte chemotaxis  regulation of cardiac muscle contraction by regulation of the release of sequestered calcium ion  positive regulation of osteoclast differentiation  mammary gland alveolus development  negative regulation of canonical Wnt receptor signaling pathway  positive regulation of epithelial cell migration  regulation of vasoconstriction  cytokine-mediated signaling pathway  monocyte differentiation  retinal ganglion cell axon guidance  gamma-aminobutyric acid signaling pathway  negative regulation of cyclin-dependent protein serine/threonine kinase activity  cell cycle  positive regulation vascular endothelial growth factor production  synaptic transmission, glutamatergic  positive regulation of cardiac muscle cell proliferation  regulation of transcription from RNA polymerase II promoter  response to radiation  membrane depolarization  positive regulation of NF-kappaB transcription factor activity  endochondral ossification  central nervous system development  protein complex assembly  positive regulation of phosphatidylinositol 3-kinase activity  regulation of calcium ion transport  endocytosis  response to virus  embryo development  substrate adhesion-dependent cell spreading  embryonic cranial skeleton morphogenesis  ion transmembrane transport  ventricular cardiac muscle tissue morphogenesis  neuromuscular junction development  melanocyte differentiation  cell division  positive regulation of gene expression  B cell receptor signaling pathway  adult walking behavior  cellular response to transforming growth factor beta stimulus  positive regulation of insulin secretion  positive regulation of interleukin-6 production  thymus development  G-protein coupled receptor signaling pathway  learning or memory  positive regulation of I-kappaB kinase/NF-kappaB cascade  defense response to Gram-positive bacterium  blood vessel remodeling  complement activation  visual learning  hippocampus development  outflow tract morphogenesis  neural crest cell migration  protein homooligomerization  positive regulation of tolerance induction to self antigen  positive regulation of B cell tolerance induction  regulation of mesenchymal cell proliferation  positive regulation of activation of Janus kinase activity  regulation of macrophage derived foam cell differentiation  satellite cell commitment  endosome transport via multivesicular body sorting pathway  positive regulation of mononuclear cell proliferation  calcium-mediated signaling using extracellular calcium source  protein localization to T-tubule  low-density lipoprotein particle receptor biosynthetic process  mucosal-associated lymphoid tissue development  negative regulation of elastin biosynthetic process  skeletal muscle organ development  mammary gland fat development  regulation of branching involved in salivary gland morphogenesis by extracellular matrix-epithelial cell signaling  regulation of cholesterol transporter activity  cardiac muscle tissue regeneration  cerebellum vasculature morphogenesis  negative regulation of interleukin-6-mediated signaling pathway  interleukin-27-mediated signaling pathway  positive regulation of histone H4-K20 methylation  atrial cardiac muscle cell to AV node cell communication  negative regulation of protein kinase C signaling cascade  positive regulation of calcium ion transmembrane transporter activity  positive regulation of macrophage colony-stimulating factor production  regulation of smooth muscle cell-matrix adhesion  positive regulation of leukocyte apoptotic process  positive regulation of histone H3-K9 acetylation  positive regulation of histone H4-K16 acetylation  positive regulation of cellular glucuronidation  blood vessel development  collagen fibril organization  cell maturation  fibroblast growth factor receptor signaling pathway  Wnt receptor signaling pathway  embryonic skeletal system morphogenesis  transport  cellular response to stress  ion transport  excretion  negative regulation of cell proliferation  skeletal muscle tissue development  positive regulation of protein ubiquitination  negative regulation of neuron differentiation  fatty acid biosynthetic process  transcription initiation from RNA polymerase II promoter  apoptotic process  neurotransmitter secretion  cell morphogenesis  regulation of cyclin-dependent protein serine/threonine kinase activity  peptidyl-tyrosine phosphorylation  columnar/cuboidal epithelial cell development  hepatic immune response  developmental process involved in reproduction  primary heart field specification  sinoatrial valve morphogenesis  glycosylceramide metabolic process  galactosylceramide biosynthetic process  adenylate cyclase-inhibiting G-protein coupled glutamate receptor signaling pathway  regulation of cardiac muscle contraction by calcium ion signaling  regulation of phosphatidylcholine catabolic process  central nervous system morphogenesis  regulation of tumor necrosis factor production  positive regulation of interleukin-1 alpha production  T-tubule organization  positive regulation of homotypic cell-cell adhesion  histone H3-T6 phosphorylation  TRAM-dependent toll-like receptor 4 signaling pathway  cellular response to trichostatin A  protein localization to M-band  nerve growth factor signaling pathway  positive regulation of odontogenesis of dentin-containing tooth  progesterone secretion  cellular pigment accumulation  glycerol-3-phosphate catabolic process  negative regulation of centriole replication  plasma membrane to endosome transport  peripheral nervous system neuron axonogenesis  negative regulation of negative chemotaxis  positive regulation of corticotropin-releasing hormone secretion  regulation of mitochondrial depolarization  regulation of synaptic activity  regulation of sarcomere organization  bronchus morphogenesis  mammary gland morphogenesis  regulation of wound healing  positive regulation of granulocyte colony-stimulating factor production  positive regulation of fever generation by positive regulation of prostaglandin secretion  TNFSF11-mediated signaling pathway  positive regulation of ERK1 and ERK2 cascade via TNFSF11-mediated signaling  T-helper 17 cell differentiation  regulation of SA node cell action potential  membrane depolarization involved in regulation of SA node cell action potential  negative regulation of protein homodimerization activity  positive regulation of potassium ion transmembrane transporter activity  regulation of calcium ion transmembrane transporter activity  positive regulation of skeletal muscle cell differentiation  malonyl-CoA biosynthetic process  retinoid metabolic process  phototransduction, visible light  forebrain development  inner ear morphogenesis  vasculogenesis  palate development  transmembrane receptor protein tyrosine kinase signaling pathway  response to nutrient  positive regulation of neuron differentiation  positive regulation of adaptive immune response  phospholipase C-activating G-protein coupled acetylcholine receptor signaling pathway  muscle cell fate determination  regulation of synaptic growth at neuromuscular junction  dosage compensation by inactivation of X chromosome  response to fructose stimulus  negative regulation of glucose transport  negative regulation of receptor biosynthetic process  gamma-aminobutyric acid secretion  visceral motor neuron differentiation  olfactory bulb interneuron development  locomotion involved in locomotory behavior  negative regulation of collagen biosynthetic process  extracellular matrix-cell signaling  norepinephrine metabolic process  chordate embryonic development  positive regulation of skeletal muscle tissue regeneration  CTP salvage  regulation of megakaryocyte differentiation  negative regulation of complement activation  negative regulation of striated muscle contraction  cAMP metabolic process  myoblast fate commitment  sensory system development  positive regulation of B cell receptor signaling pathway  multicellular organismal water homeostasis  negative regulation of histone H3-K4 methylation  positive regulation of histone H3-K9 methylation  positive regulation of dopamine uptake involved in synaptic transmission  synaptic transmission, GABAergic  response to low-density lipoprotein particle stimulus  vestibular reflex  rhythmic synaptic transmission  smooth muscle contraction involved in micturition  cardiac muscle cell myoblast differentiation  trachea formation  retinal blood vessel morphogenesis  renal tubule morphogenesis  apoptotic process involved in luteolysis  positive regulation of mucus secretion  sarcoplasmic reticulum calcium ion transport  cholesterol import  D-aspartate import  cellular response to carbohydrate stimulus  cellular response to oxygen levels  positive regulation of cell proliferation in bone marrow  regulation of branch elongation involved in ureteric bud branching  glomerulus morphogenesis  nephron tubule epithelial cell differentiation  SA node cell to atrial cardiac muscle cell communication  positive regulation of STAT protein import into nucleus  epithelial cell proliferation involved in renal tubule morphogenesis  positive regulation of protein kinase B signaling cascade  blood coagulation  canonical Wnt receptor signaling pathway  glucose homeostasis  sodium ion transport  regulation of insulin secretion  protein localization to paranode region of axon  positive regulation of T cell tolerance induction  regulation of secondary heart field cardioblast proliferation  acetyl-CoA metabolic process  regulation of Notch signaling pathway  regulation of smooth muscle cell migration  trigeminal nerve development  regulation of vascular endothelial growth factor receptor signaling pathway  peroxisome proliferator activated receptor signaling pathway  non-canonical Wnt receptor signaling pathway via JNK cascade  regulation of DNA methylation  glucose import  positive regulation of NK T cell differentiation  response to nitrosative stress  myoblast migration  ventricular cardiac myofibril development  negative regulation of interferon-gamma-mediated signaling pathway  muscle tissue morphogenesis  mammary placode formation  cardiac cell fate determination  retina vasculature morphogenesis in camera-type eye  cardiac conduction  protein localization to endoplasmic reticulum  positive regulation of ureteric bud formation  positive regulation of protein localization to cell surface  positive regulation of macrophage apoptotic process  positive regulation of behavioral fear response  positive regulation of cation channel activity  cellular response to hypoxia  Fc-gamma receptor signaling pathway involved in phagocytosis  positive regulation of angiogenesis  cell-cell adhesion  potassium ion transport  muscle organ development  neutrophil mediated immunity  regulation of germinal center formation  immune response-regulating cell surface receptor signaling pathway  atrial septum development  brainstem development  cysteine metabolic process  sphingomyelin biosynthetic process  NADH metabolic process  response to xenobiotic stimulus  negative regulation of sequestering of triglyceride  smooth muscle cell migration  carbon dioxide transport  axon midline choice point recognition  olfactory bulb interneuron differentiation  cell cycle process  regulation of T cell differentiation in thymus  response to lipid  negative regulation of translation involved in gene silencing by miRNA  locomotion  hyperosmotic salinity response  regulation of ion transport  regulation of transcription from RNA polymerase II promoter in response to oxidative stress  UMP salvage  positive regulation of monocyte differentiation  positive regulation of fatty acid oxidation  mitochondrion distribution  regulation of phagocytosis  positive regulation of lipid catabolic process  negative regulation of lipoprotein lipase activity  positive regulation of keratinocyte migration  L-glutamate import  negative regulation of synapse assembly  mammary duct terminal end bud growth  renal water absorption  cellular response to parathyroid hormone stimulus  cellular response to fluid shear stress  cellular response to indole-3-methanol  cell communication by electrical coupling involved in cardiac conduction  DNA repair  anatomical structure morphogenesis  intracellular protein kinase cascade  aging  neurotransmitter uptake  complement activation, lectin pathway  macrophage activation involved in immune response  cardiac ventricle formation  centrosome cycle  common-partner SMAD protein phosphorylation  Wnt receptor signaling pathway, calcium modulating pathway  positive regulation of alkaline phosphatase activity  negative regulation of cholesterol storage  long-chain fatty acid transport  spinal cord motor neuron cell fate specification  cerebral cortex neuron differentiation  cellular phosphate ion homeostasis  paranodal junction assembly  negative regulation of interleukin-8 production  positive regulation of granulocyte macrophage colony-stimulating factor production  negative regulation of Rho GTPase activity  negative regulation of heart contraction  positive regulation of skeletal muscle tissue development  semicircular canal morphogenesis  regulation of steroid biosynthetic process  regulation of smooth muscle cell differentiation  negative regulation of stress fiber assembly  negative regulation of histone H3-K9 methylation  negative regulation of cardiac muscle cell proliferation  lung lobe morphogenesis  positive regulation of protein homodimerization activity  positive regulation of nuclear-transcribed mRNA catabolic process, deadenylation-dependent decay  protein glycosylation  negative regulation of acute inflammatory response  postreplication repair  positive regulation of intracellular protein kinase cascade  positive regulation of fibroblast migration  neural crest cell differentiation  transmission of nerve impulse  neuronal action potential propagation  negative regulation of epithelial cell differentiation  locomotory exploration behavior  monocyte activation  mesenchymal cell differentiation  regulation of release of sequestered calcium ion into cytosol  reduction of cytosolic calcium ion concentration  negative regulation of telomerase activity  ventricular cardiac muscle cell differentiation  inner ear receptor cell development  positive regulation of nuclear-transcribed mRNA poly(A) tail shortening  regulation of ventricular cardiac muscle cell membrane depolarization  cartilage morphogenesis  response to cholesterol  cellular response to ethanol  protein K6-linked ubiquitination  regulation of atrial cardiac muscle cell action potential  positive regulation of cardiac muscle cell differentiation  regulation of cell shape  mitochondrial genome maintenance  morphogenesis of a branching structure  regulation of transcription from RNA polymerase III promoter  neuron remodeling  growth hormone secretion  regulation of ossification  intestinal cholesterol absorption  positive regulation of cellular metabolic process  positive regulation of interleukin-1 beta production  low-density lipoprotein particle clearance  wound healing, spreading of epidermal cells  endothelial cell differentiation  positive regulation of macrophage differentiation  regulation of osteoclast differentiation  Peyer's patch development  cellular response to dexamethasone stimulus  metanephric mesenchyme development  negative regulation of cell growth | 4  9  6  11  9  9  5  9  5  6  4  7  4  4  4  6  6  3  3  2  4  7  3  3  6  2  7  4  5  3  9  4  4  10  4  3  3  2  2  6  3  6  3  4  3  5  2  3  4  2  2  2  6  3  2  5  3  3  2  3  4  4  3  3  2  2  2  2  2  2  3  2  2  4  2  2  2  2  4  2  2  2  4  2  2  3  2  3  3  2  2  3  3  3  2  2  3  2  2  2  4  3  2  2  2  2  2  2  4  2  3  2  2  2  2  2  2  2  3  1  1  1  1  1  1  1  1  1  1  1  1  1  1  1  1  1  1  1  1  1  1  1  1  1  1  1  1  1  1  1  2  2  2  3  3  2  4  2  3  2  4  2  2  2  2  3  5  2  2  2  2  1  1  1  1  1  1  1  1  1  1  1  1  1  1  1  1  1  1  1  1  1  1  1  1  1  1  1  1  1  1  1  1  1  1  1  1  1  1  1  1  1  1  1  1  1  1  1  2  2  2  2  2  2  22  2  1  1  1  1  1  1  1  1  1  1  1  1  1  1  1  1  1  1  1  1  1  1  1  1  1  1  1  1  1  1  1  1  1  1  1  1  1  1  1  1  1  1  1  1  1  1  1  1  1  1  1  1  2  4  2  2  2  2  1  1  1  1  1  1  1  1  1  1  1  1  1  1  1  1  1  1  1  1  1  1  1  1  1  1  1  1  2  2  2  2  2  2  1  1  1  1  1  1  1  1  1  1  1  1  1  1  1  1  1  1  1  1  1  1  1  1  1  1  1  1  1  1  1  1  1  1  1  1  1  1  3  2  2  2  1  1  1  1  1  1  1  1  1  1  1  1  1  1  1  1  1  1  1  1  1  1  1  1  1  1  1  1  2  1  1  1  1  1  1  1  1  1  1  1  1  1  1  1  1  1  1  1  1  1  1  1  1  2  1  1  1  1  1  1  1  1  1  1  1  1  1  1  1  1  1  2 | 10.121  7.745  7.667  7.644  7.490  6.882  6.575  6.384  6.371  6.096  5.831  5.819  5.376  5.248  5.158  4.994  4.859  4.841  4.777  4.622  4.572  4.565  4.501  4.501  4.428  4.321  4.308  4.306  4.276  4.233  4.184  4.139  4.047  4.014  3.988  3.946  3.914  3.778  3.778  3.754  3.738  3.711  3.685  3.662  3.659  3.658  3.654  3.634  3.570  3.545  3.448  3.448  3.439  3.364  3.362  3.308  3.285  3.285  3.283  3.266  3.230  3.230  3.230  3.159  3.144  3.144  3.082  3.082  3.082  3.082  3.076  3.025  3.025  2.973  2.971  2.971  2.920  2.920  2.914  2.872  2.827  2.827  2.773  2.743  2.743  2.681  2.666  2.658  2.636  2.630  2.630  2.614  2.614  2.614  2.596  2.596  2.581  2.563  2.531  2.471  2.466  2.450  2.443  2.415  2.415  2.388  2.388  2.388  2.371  2.362  2.340  2.337  2.313  2.313  2.313  2.313  2.289  2.266  2.256  2.246  2.246  2.246  2.246  2.246  2.246  2.246  2.246  2.246  2.246  2.246  2.246  2.246  2.246  2.246  2.246  2.246  2.246  2.246  2.246  2.246  2.246  2.246  2.246  2.246  2.246  2.246  2.246  2.246  2.246  2.246  2.243  2.243  2.243  2.232  2.224  2.222  2.193  2.179  2.162  2.159  2.157  2.101  2.101  2.064  2.047  2.028  2.008  1.996  1.980  1.964  1.948  1.945  1.945  1.945  1.945  1.945  1.945  1.945  1.945  1.945  1.945  1.945  1.945  1.945  1.945  1.945  1.945  1.945  1.945  1.945  1.945  1.945  1.945  1.945  1.945  1.945  1.945  1.945  1.945  1.945  1.945  1.945  1.945  1.945  1.945  1.945  1.945  1.945  1.945  1.945  1.945  1.945  1.945  1.945  1.945  1.945  1.945  1.945  1.932  1.917  1.917  1.917  1.832  1.832  1.819  1.780  1.780  1.769  1.769  1.769  1.769  1.769  1.769  1.769  1.769  1.769  1.769  1.769  1.769  1.769  1.769  1.769  1.769  1.769  1.769  1.769  1.769  1.769  1.769  1.769  1.769  1.769  1.769  1.769  1.769  1.769  1.769  1.769  1.769  1.769  1.769  1.769  1.769  1.769  1.769  1.769  1.769  1.769  1.769  1.769  1.769  1.769  1.769  1.769  1.769  1.769  1.769  1.769  1.769  1.767  1.751  1.707  1.684  1.673  1.662  1.644  1.644  1.644  1.644  1.644  1.644  1.644  1.644  1.644  1.644  1.644  1.644  1.644  1.644  1.644  1.644  1.644  1.644  1.644  1.644  1.644  1.644  1.644  1.644  1.644  1.644  1.644  1.644  1.640  1.609  1.589  1.569  1.550  1.550  1.548  1.548  1.548  1.548  1.548  1.548  1.548  1.548  1.548  1.548  1.548  1.548  1.548  1.548  1.548  1.548  1.548  1.548  1.548  1.548  1.548  1.548  1.548  1.548  1.548  1.548  1.548  1.548  1.548  1.548  1.548  1.548  1.548  1.548  1.548  1.548  1.548  1.548  1.537  1.504  1.495  1.495  1.469  1.469  1.469  1.469  1.469  1.469  1.469  1.469  1.469  1.469  1.469  1.469  1.469  1.469  1.469  1.469  1.469  1.469  1.469  1.469  1.469  1.469  1.469  1.469  1.469  1.469  1.469  1.469  1.460  1.402  1.402  1.402  1.402  1.402  1.402  1.402  1.402  1.402  1.402  1.402  1.402  1.402  1.402  1.402  1.402  1.402  1.402  .402  1.402  1.402  1.402  1.402  1.402  1.394  1.345  1.345  1.345  1.345  1.345  1.345  1.345  1.345  1.345  1.345  1.345  1.345  1.345  1.345  1.345  1.345  1.345  1.313 | 0.000  0.000  0.000  0.000  0.000  0.000  0.000  0.000  0.000  0.000  0.000  0.000  0.000  0.000  0.000  0.001  0.001  0.001  0.001  0.001  0.001  0.001  0.001  0.001  0.001  0.002  0.002  0.002  0.002  0.002  0.002  0.002  0.002  0.002  0.002  0.003  0.003  0.004  0.004  0.004  0.004  0.004  0.004  0.004  0.004  0.004  0.004  0.004  0.005  0.005  0.006  0.006  0.006  0.007  0.007  0.007  0.008  0.008  0.008  0.008  0.008  0.008  0.008  0.009  0.009  0.009  0.010  0.010  0.010  0.010  0.010  0.011  0.011  0.012  0.012  0.012  0.013  0.013  0.013  0.014  0.015  0.015  0.017  0.018  0.018  0.021  0.021  0.021  0.022  0.022  0.022  0.022  0.022  0.022  0.022  0.022  0.023  0.024  0.025  0.029  0.029  0.030  0.030  0.031  0.031  0.032  0.032  0.032  0.032  0.032  0.032  0.032  0.032  0.032  0.032  0.032  0.032  0.032  0.032  0.032  0.032  0.032  0.032  0.032  0.032  0.032  0.032  0.032  0.032  0.032  0.032  0.032  0.032  0.032  0.032  0.032  0.032  0.032  0.032  0.032  0.032  0.032  0.032  0.032  0.032  0.032  0.032  0.032  0.032  0.032  0.032  0.032  0.032  0.032  0.033  0.033  0.035  0.036  0.037  0.037  0.037  0.041  0.041  0.044  0.044  0.044  0.044  0.044  0.044  0.044  0.044  0.044  0.044  0.044  0.044  0.044  0.044  0.044  0.044  0.044  0.044  0.044  0.044  0.044  0.044  0.044  0.044  0.044  0.044  0.044  0.044  0.044  0.044  0.044  0.044  0.044  0.044  0.044  0.044  0.044  0.044  0.044  0.044  0.044  0.044  0.044  0.044  0.044  0.044  0.044  0.044  0.044  0.044  0.044  0.044  0.044  0.044  0.044  0.045  0.046  0.046  0.046  0.052  0.052  0.052  0.052  0.052  0.052  0.052  0.052  0.052  0.052  0.052  0.052  0.052  0.052  0.052  0.052  0.052  0.052  0.052  0.052  0.052  0.052  0.052  0.052  0.052  0.052  0.052  0.052  0.052  0.052  0.052  0.052  0.052  0.052  0.052  0.052  0.052  0.052  0.052  0.052  0.052  0.052  0.052  0.052  0.052  0.052  0.052  0.052  0.052  0.052  0.052  0.052  0.052  0.052  0.052  0.052  0.052  0.052  0.054  0.059  0.062  0.062  0.062  0.062  0.062  0.062  0.062  0.062  0.062  0.062  0.062  0.062  0.062  0.062  0.062  0.062  0.062  0.062  0.062  0.062  0.062  0.062  0.062  0.062  0.062  0.062  0.062  0.062  0.062  0.062  0.062  0.062  0.066  0.067  0.067  0.067  0.067  0.067  0.067  0.067  0.067  0.067  0.067  0.067  0.067  0.067  0.067  0.067  0.067  0.067  0.067  0.067  0.067  0.067  0.067  0.067  0.067  0.067  0.067  0.067  0.067  0.067  0.067  0.067  0.067  0.067  0.067  0.067  0.067  0.067  0.067  0.067  0.067  0.067  0.067  0.069  0.074  0.074  0.074  0.074  0.074  0.074  0.074  0.074  0.074  0.074  0.074  0.074  0.074  0.074  0.074  0.074  0.074  0.074  0.074  0.074  0.074  0.074  0.074  0.074  0.074  0.074  0.074  0.074  0.074  0.074  0.074  0.076  0.081  0.081  0.081  0.081  0.081  0.081  0.081  0.081  0.081  0.081  0.081  0.081  0.081  0.081  0.081  0.081  0.081  0.081  0.081  0.081  0.081  0.081  0.081  0.081  0.083  0.089  0.089  0.089  0.089  0.089  0.089  0.089  0.089  0.089  0.089  0.089  0.089  0.089  0.089  0.089  0.089  0.089  0.092 |
